# RGS6 drives cardiomyocyte death following nucleolar stress by suppressing Nucleolin/miRNA-21

**DOI:** 10.1186/s12967-024-04985-3

**Published:** 2024-02-26

**Authors:** Abhishek Singh Sengar, Manish Kumar, Chetna Rai, Sreemoyee Chakraborti, Dinesh Kumar, Pranesh Kumar, Sukhes Mukherjee, Kausik Mondal, Adele Stewart, Biswanath Maity

**Affiliations:** 1 Centre of Biomedical Research (CBMR), SGPGI Campus, Raebareli Road, Lucknow, Uttar Pradesh 226014 India; 2 Forensic Science Laboratory, Department of Home and Hill Affairs, Kolkata, West Bengal 700037 India; 3 Institute of Pharmaceutical Science, University of Lucknow, Lucknow, Uttar Pradesh 226007 India; 4 Biochemistry, AIIMS Bhopal, Saket Nagar, Bhopal, Madhya Pradesh 462026 India; 5 Zoology, University of Kalyani, Nadia, West Bengal 741235 India; 6 Biomedical Science, Florida Atlantic University, Jupiter, FL 33458 USA

**Keywords:** Cardiotoxicity, G-protein, RGS proteins, Chemotherapy, Nucleolar stress

## Abstract

**Background:**

Prior evidence demonstrated that Regulator of G protein Signaling 6 (RGS6) translocates to the nucleolus in response to cytotoxic stress though the functional significance of this phenomenon remains unknown.

**Methods:**

Utilizing in vivo gene manipulations in mice, primary murine cardiac cells, human cell lines and human patient samples we dissect the participation of a RGS6-nucleolin complex in chemotherapy-dependent cardiotoxicity.

**Results:**

Here we demonstrate that RGS6 binds to a key nucleolar protein, Nucleolin, and controls its expression and activity in cardiomyocytes. In the human myocyte AC-16 cell line, induced pluripotent stem cell derived cardiomyocytes, primary murine cardiomyocytes, and the intact murine myocardium tuning RGS6 levels via overexpression or knockdown resulted in diametrically opposed impacts on Nucleolin mRNA, protein, and phosphorylation.RGS6 depletion provided marked protection against nucleolar stress-mediated cell death in vitro, and, conversely, RGS6 overexpression suppressed ribosomal RNA production, a key output of the nucleolus, and triggered death of myocytes. Importantly, overexpression of either Nucleolin or Nucleolin effector miRNA-21 counteracted the pro-apoptotic effects of RGS6. In both human and murine heart tissue, exposure to the genotoxic stressor doxorubicin was associated with an increase in the ratio of RGS6/Nucleolin. Preventing RGS6 induction via introduction of RGS6-directed shRNA via intracardiac injection proved cardioprotective in mice and was accompanied by restored Nucleolin/miRNA-21 expression, decreased nucleolar stress, and decreased expression of pro-apoptotic, hypertrophy, and oxidative stress markers in heart.

**Conclusion:**

Together, these data implicate RGS6 as a driver of nucleolar stress-dependent cell death in cardiomyocytes via its ability to modulate Nucleolin. This work represents the first demonstration of a functional role for an RGS protein in the nucleolus and identifies the RGS6/Nucleolin interaction as a possible new therapeutic target in the prevention of cardiotoxicity.

**Supplementary Information:**

The online version contains supplementary material available at 10.1186/s12967-024-04985-3.

## Introduction

Ribosome biogenesis wherein ribosomal RNA (rRNA) is generated, processed, assembled with ribosomal proteins, and exported to the cytoplasm is a highly evolutionarily conserved biological process essential to life [1]. In eukaryotic cells this process occurs in a specialized nuclear sub-compartment, the nucleolus, which contains ribosomal DNA (rDNA) and ribonucleoproteins necessary for rDNA transcription [2]. Nucleolar architecture dynamically reacts to the demand for ribosome production, and, as a result, it is becoming increasingly clear that the cellular impacts of genotoxic and oxidative stress as well as nutrient deprivation and hypoxia are reflected in the nucleolus, which also participates in stress responsive signal transduction [3, 4]. Importantly, several disease states are associated with disruptions in nucleolar function including heart disease [5], which remains the leading cause of death worldwide. Indeed, the hearts of human patients with ischemic or dilated cardiomyopathy displayed increased nucleolar size, fibrillar centers, perinucleolar chromatin, and dense fibrillar components indicative of ongoing nucleolar stress [6]. However, a comprehensive understanding of the role of nucleolar dysfunction in the pathogenesis of heart disease or delineation of the molecular mechanisms linking cardiotoxic stressors to the nucleolus has yet to be achieved. Doxorubicin, an anthracycline, anti-tumor drug is one of such stressors which is associated with cardiotoxicity that limits the use of this drug in long term [7].

Nucleolar proteins, which vastly outnumber those necessary for ribosome biogenesis [8], shuttle in and out of the nucleolus under certain stress conditions [9–11]. In cardiac cell types, nucleolar proteins Nucleostemin and Nucleophosmin promote cell survival and proliferation and their depletion can induce cell death [12, 13]. Similarly, decreased expression of Nucleolin has been observed in mouse heart following ischemia–reperfusion injury and during hypertrophy and heart failure [14, 15]. Nucleolin knockdown in the myocardium was associated with increased mortality and compromised systolic and diastolic cardiac function [15], while Nucleolin overexpression is cardioprotective [14, 16]. Though the underlying mechanisms remain unclear, Nucleolin has been implicated in hypoxia- or H_2_O_2_-induced cardiomyocyte death [14, 17] and recent evidence has linked the anti-apoptotic actions of Nucleolin to regulation of cardiac microRNA-21 (miRNA-21) [16, 18, 19], whose targets include several genes involved in cell death signaling such as *PTEN*, *BCL2*, *FASL*, *TP63*, and *PPARA* [20]. Importantly, Nucleolin expression correlates with left ventricular function in patients with ischemic cardiomyopathy [6]. Thus, in cardiac disease states, nucleolar proteins may play a key role in balancing repair and regeneration with elimination of functionally compromised cells.

The canonical function of Regulators of G protein Signaling (RGS) proteins is in stabilization of the transition state of GTP hydrolysis by the Gα subunit of the heterotrimeric G protein complex thereby accelerating termination of G protein signal transduction [21]. However, recent efforts to characterize the diverse functions of the nearly 40 members of this protein family have revealed novel, G protein independent functions for RGS proteins. For example, the R7 family member RGS6 is potently pro-apoptotic in myocytes and other cell types [22–25]. Indeed, RGS6^−/−^ mice are protected against the cardiotoxic impacts of the chemotherapeutic drug doxorubicin [23], known to induce nucleolar stress in vivo [13]. RGS6 is also required for doxorubicin-dependent activation of ATM serine/threonine kinase (ATM) and p53 [23, 24], and RGS6 forms a co-precipitable complex with both ATM [25] and the nuclear transcriptional regulator DNA methyltransferase 1 (DNMT1) [26] suggesting that RGS6 may localize to the nucleus in response to genotoxic stress. In fact, a prior report noted that mild heat, proteotoxic stress, and inhibition of rDNA transcription induce nucleolar trafficking and accumulation of RGS6 [27] though the functional significance of this phenomenon remains unknown. However, the observation that RGS6 binding partner ATM also triggers nucleolar reorganization [28] lends credence to the idea that nucleolar accumulation of RGS6 might play a critical role in cellular stress signaling.

Here, we demonstrate that RGS6 binds to nucleolar protein Nucleolin and that the ability of RGS6 to suppress Nucleolin phosphorylation and expression and control miRNA-21 levels underlies, at least in part, the pro-apoptotic actions of RGS6 in cardiomyocytes and the murine myocardium.

## Materials and methods

### Reagents

A complete list of reagents utilized to generate the data in the manuscript can be found in Additional file 1: Table S1.

### Mice

Male Swiss albino mice (25–30 g) were reared on a balanced laboratory diet usually given tap water and food ad libitum throughout the study as per NIN, Hyderabad, India, and were kept at 20 ± 2 °C, 65% to 70% humidity, on a 12/12-h day/night cycle. Animals were handled following International Animal Ethics Committee Guidelines. 12–14-week-old animals were subjected to chronic chemotherapeutic drug regimens. Unless otherwise noted, experiments have been performed using animals between 2–3 months of age. The entire study was performed & analyzed by a blinded observer. Exact numbers of animals used for each experiment can be found in the Data Supplement. Unless otherwise noted, experimental mice were euthanized using inhalation anesthesia followed by cervical dislocation and disposed of following CCSEA guidelines.

### Doxorubicin treatment

To approximate human chemotherapy treatment we utilized a chronic, low-dose doxorubicin treatment paradigm [29, 30]. Briefly, mice received multiple doses of doxorubicin (cumulative dose of 45 mg/kg i.p.; 9 mg/kg every other week) or saline over a period of 10 weeks. One week after the final drug dose mice were euthanized by cervical dislocation and blood/multiple tissues were collected for downstream analysis.

### RGS6 knockdown or over expression in vivo

To achieve Cardiac RGS6 knockdown 1-week-old wild type (WT) mice received intra-cardiac single dose of at least 5X 10^8^ Scramble or RGS6-targeted lentiviral vector ShRNA particles (Santacruz Biotechnology, Paso Robles, CA, USA) essentially as previously described [29] in a 40 µl volume and packaged for delivery with invivofectamine 3.0 (ThermoFisher Scientific, Waltham, MA, USA). AC-16 cells were used for the generation of lentiviral particles as manufacturers’ protocol (Santacruz Biotechnology, Paso Robles, CA, USA).Immunoblotting was performed to assess the efficiency of in vivo delivered shRNA. Following shRNA administration, body weight (1X/week) and food intakes (1–2X/week) were monitored. No alterations in animal weight, food intake or general wellbeing were noted (data not shown). Doxorubicin treatment (as above) was initiated in a subset of animals for 8–10 weeks following intra-cardiac injection.

In a separate experiment, a lentiviral construct encoding murine RGS6 or vector-only control was introduced to the murine heart via intracardiac injection (n = 6/group) when mice were aged 8–10 weeks old. Mice were euthanized by cervical dislocation and blood/multiple tissues were collected for downstream analysis 10 weeks after viral transduction. Viral constructs utilized for mRGS6 overexpression in vivo have been previously described [25]. Lentiviral particles were generated in AC-16 cells and 70 μL of lentivirus containing 2 × 10^8^ particles of either mRGS6-Lenti or a control empty vector virus were packaged for delivery with invivofectamine 3.0 (Thermo Fisher Scientific) and administered via intra-cardiac injection.

### Cell isolation and culture

Primary ventricular cardiomyocytes (VCM) were isolated from 8–10-week-old adult mice according to a published protocol [31]. The human cardiomyocyte cell line AC-16 (Merck, Darmstadt, Germany) was cultured in DMEM and 10% FBS (Gibco, Waltham, MA, USA) in a 37 °C incubator at 5% CO_2_. Human induced pluripotent stem cell derived cardiomyocytes (cell artis cardiomyocytes, Takara Bio, San Jose, CA, USA) were cultured in cell artis CM culture base medium and 10% FBS (Gibco) in a 37 °C incubator at 5% CO_2_and < 90% humidity. Cells were transduced with vectors encoding scramble, RGS6-, or Nucleolin-targeting shRNA (Santacruz Biotechnology) or viral overexpression constructs for RGS6 (full length, deletion and point mutation constructs) or Nucleolin. miRNA21 OE was achieved using Lipofectamine 2000 (Thermo Fisher Scientific).Catalog information for purchased cell lines is available in Additional file 1: Table S2.

### Immunohistochemistry

Immunohistochemical staining of both mouse and human tissue sections was performed as per a standard protocol [32]. For RGS6, Nucleolin staining, 7–10 sections were stained from each animal with 5 pictures randomly selected from each slide for quantification using Image J.

### Cloning and construct generation

We have previously described generation of constructs encoding full-length RGS6 and truncation mutants [25]. Here, we utilized a similar approach to clone human or mouse Nucleolin from human blood or mouse braincDNAinto the pMD-20T vector later sub-cloning into the pLenti CMV Puro DEST cloning vector (Add gene, Watertown, MA, USA). The miRNA-21 overexpression constructs for both the human and mouse sequence were cloned into the pmR-ZsGreen1 vector (Takara Bio) after obtaining the pre-miRNA-21 sequence from genomic DNA. Each construct contains the miRNA-21 sequence along with upstream and downstream sequence extended up to 100–150 bp to ensure efficient processing by Drosha. A negative control construct was also generated by cloning a nonspecific sequence. All the information pertaining to primers utilized in the study for construct preparation has been included in Additional file 1: Table S5.

### Drug treatment of cultured cells

Cells were allowed to reach 80–85% confluence in standard culture media prior to drug treatments. The media was then replaced with serum-free DMEM, and doxorubicin (3 µM) or actinomycin D (5 µM) were then added and FBS added back to the media after 6 h. After 16 (actinomycin D) or 18 h (doxorubicin) cells were washed twice with PBS and harvested by centrifugation for downstream analyses. 50 nM anti-miRNA-21(miRNA-21i; AcceGen, Fairfield, NJ, USA)were introduced into the cells by using Lipofectamine2000 (Thermo Fisher Scientific) according to the manufacturer’s protocol. The mouse sequence was used for experiments in primary VCM and the human sequence for AC-16 cell experiments. The transfection efficiency was assessed by qPCR.

### Immunoblotting

Tissues were rapidly dissected from mice and flash frozen in liquid nitrogen. Cells were washed in PBS and harvested via centrifugation. Tissue homogenates and cell lysates were prepared in RIPA buffer containing protease and phosphatase inhibitors (Sigma, St. Louis, MO, USA), quantified, and probed as previously described [32]. Twenty µg of protein per sample was subjected to SDS-PAGE and immunoblotting using standard techniques. Immunoblots were developed using chemiluminescence with HRP-labeled secondary antibodies and visualized using the UVP Chemstudio system (Analytik Jena, Jena, Germany). Antibody dilution and catalog information can be found in Additional file 1: Table S3. Densitometric quantification of western blots was performed utilizing Image J software (NIH). Protein expression was normalized to loading control (β-Actin) and expressed relative to control conditions.

### Quantitative PCR

RNA was isolated from the tissue or cells using the TRIzol reagent (Invitrogen, Waltham, MA, USA) and concentration as well as purity (^A^260/^A^280 ratio) was measured using a Nanodrop spectrophotometer (BioTEK, Winooski, VT, USA).cDNA was generated from RNA the Verso cDNA synthesis kit (ThermoFisherScientific) and cDNA was stored at − 20 °C. Realtime PCR assays were performed using a SYBR green master mix (Bio-Rad, Hercules, CA, USA) and QuantStudio3 thermocycler (ThermoFisher Scientific). Data was analyzed using the comparative C_T_2^−ΔΔC^_T_ method. All values were normalized against a calibrator gene (*U6* for miRNA-21 and *GAPDH* for all others) and expressed relative to gene expression in control samples. Synthesis of Pre-rRNA in samples was determined by previously published protocol [13]. A complete list of the primers used is included in Additional file 1: Table S5.

### Co-immunoprecipitation (Co-IP)

AC-16 cells (3 X 10^6^) were lysed, and protein concentration measured withtheBCA protein assay. (Thermo Fisher Scientific) as per manufacturers’ protocol. 500 µg of protein was equilibrated in IP lysis buffer (50 mM Tris, 5 mM EDTA, 250 mM NaCl and 0.1% Triton X-100) with bait antibodies (anti-GFPor control mouse IgG) for 12 h on a rotor at 4 °C. 30 µl of Protein AG sepharose beads (Abcam, Waltham, MA, USA) were pre-cleared, equilibrated and then added to lysate. After a 2-h incubation, bead slurries were centrifuged and washed 3X with IP buffer. Immunocomplexes were eluted in non-reducing laemmli buffer at 95 °C and subjected to SDS-PAGE and immunoblotting with prey antibody (anti-GFP or HA).

### Measurement of ROS generation

The cell-permeable oxidation-sensitive probe, CM-H_2_DCFDA was used to estimate ROS generation in the primary or secondary cells as described previously [33]. Briefly, cells were harvested by centrifugation, washed three times with ice-cold PBS, re-suspended in PBS and incubated with 5 μM CM-H_2_DCFDA (Sigma) for 20 min at 37 °C. After incubation, cells were again washed and lysed in PBS with 1% Tween 20. ROS level was determined at the ratio of dichlorofluorescein excitation at 480 nm to emission at 530 nm. The CM-H_2_DCFDA assay is utilized as a general oxidative stress indicator and not as a detector of a specific oxidant due to known limitations of the probe [34].

### Cell viability

The MTT reduction assay was used to monitor cell viability. 6 × 10^4^ cells/well were seeded in 96 well plates with DMEM + 10% FCS. The constructs were introduced into the cells as above and cells were harvested after 36 h. The MTT (Sigma) solution was prepared at 1 mg/mL concentration in medium without phenol red, and 200 μL of MTT solution was added into each well. The cells were incubated for 2 h at 37 °C. 200 μL of DMSO was then added into each well for solubilization of the formed formazan crystals. The optical density of the wells was measured at a wavelength of 550 nm (Microplatereader, Biotek Instruments).

### ELISAs and enzymatic assays

A summary of commercially available kits used to isolate mitochondria, measure levels of mitochondrial Ca^2+^, and estimate cell death (apoptosis; cytoplasmic histone-associated DNA fragments), is available in Additional file 1: Table S4. Cells were harvested, and samples processed according to the manufacturer’s instructions.

### In-silico molecular docking and molecular dynamics (MD) simulations

To generate the protein–protein interaction model between RGS6 and Nucleolin, the alpha-fold 3D structures available on Uniprot database for human RGS6 (ID: P49758|https://www.uniprot.org/uniprotkb/P49758/entry) and Nucleolin (710 amino acids|ID: P19338|https://www.uniprot.org/uniprotkb/P19338/entry) were used. Before molecular docking experiments, the flexible unstructured N- and C-terminal fragments were removed from the alpha-fold structure of human Nucleolin protein and the truncated Nucleolin structure from Gly300-Trp644 was used for further computational work. Next, the binding modes of RGS6 in complex with Nucleolin were generated using the ZDOCK webserver application (https://zdock.umassmed.edu) [35, 36]. The molecular complex between RGS6 and Nucleolin with the highest binding energy was further evaluated for solution stability under biological conditions by performing a 127.25 ns molecular dynamics (MD) simulation in an explicit water solvent using YASARA Dynamics software (20.7.4.W.64 employing the AMBER14 force field) [37]. The MD simulation was performed as per the details and parameters described previously [38, 39]. As both proteins are large, the molecular structures were truncated prior to performing the MD simulation (RGS6 residues Met1-Arg254, Nucleolin residues Arg561-Trp644). The MD snapshots were saved after every 250 ps and a total of 510MD trajectories were generated during the MD simulations which were analysed using the YASARA macro “md_analysis.mcr”. Energy terms were calculated using AMBER14 force field parameters [40, 41]. The MD simulation and trajectory analysis was performed on a Dell Precision T7810 Tower Workstation (16 GB of RAM, 2.40 GHz Intel® Xeon® Processor E5-2630 v3 and 64 bit Windows 10 operating system). The trajectories generated after MD simulation experiments were used to estimate the root mean square deviations (RMSD) and radius of gyration (Rg), chain-wise root mean square fluctuations (RMSF), solvent accessible surface area (SASA), and hydrogen bonding between protein and solvent. For the analysis of amino acid residues present on protein–protein interaction interface, the solvent accessible surface area (SASA) calculations were performed using the InterProSurf Webserver (http://curie.utmb.edu/usercomplex.html) [42]. The residues showing significant change in the SASA value were considered most likely to be on the interaction interface.

### Statistical analysis

All the statistical analysis was performed by GraphPad Prism9 software. Data was analyzed using student’s two-tailed t-test and one- or two-way ANOVA with Sidak’s post hoc tests. Differences were considered statistically significant at *P* < 0.05.

## Results

### RGS6 binds to Nucleolin and regulates Nucleolin expression in myocytes

**Fig. 1:**
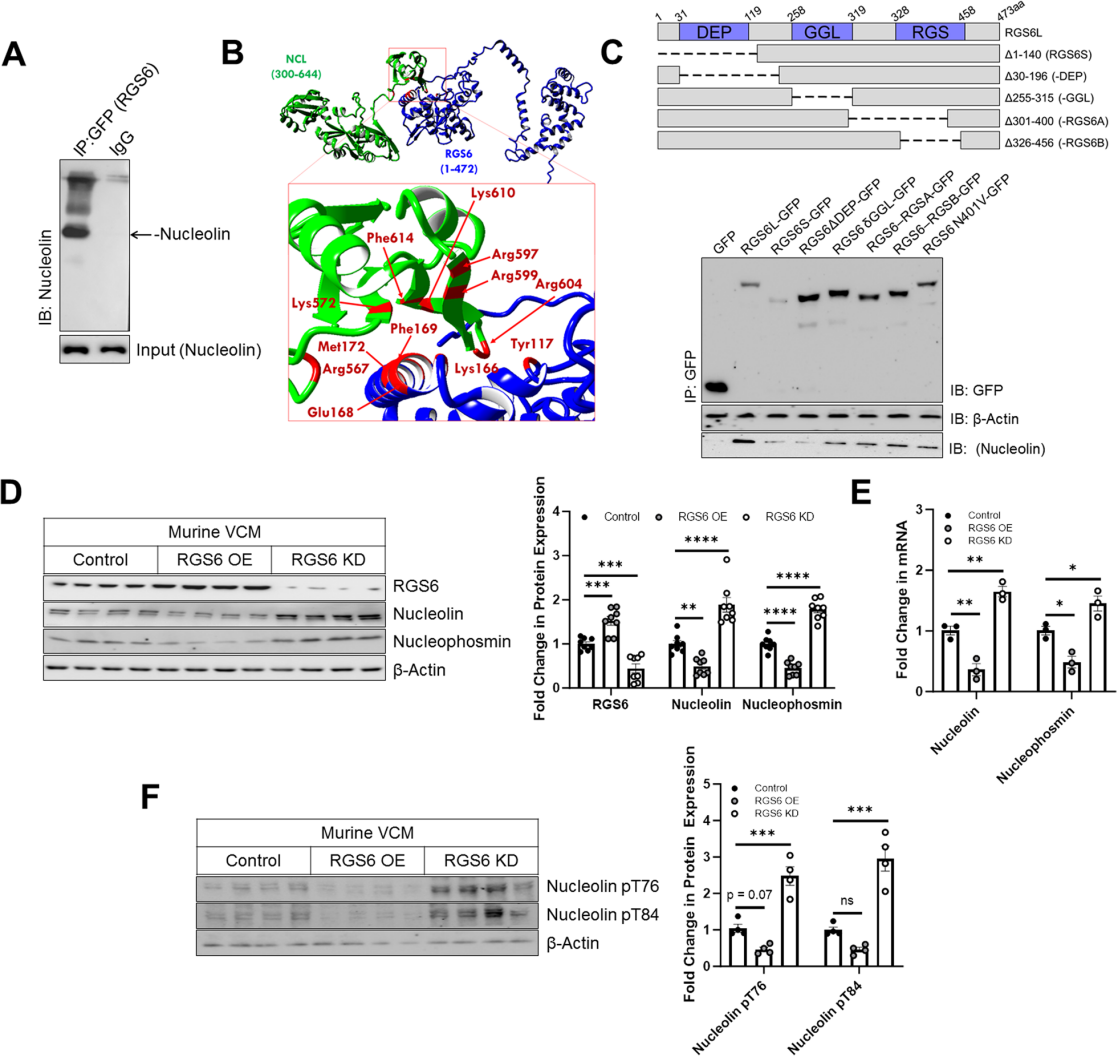
RGS6 forms a complex with Nucleolin in VCM. **A** Co-immunoprecipitation (Co-IP) of RGS6 with Nucleolin from human AC-16 cardiomyocytes. **B** In silico modeling of the RGS6-Nucleolin supports direct interaction.The best docking pose for the RGS6-Nucleolin (NCL) complex predicted using the ZDOCK webserver. The residues present on the protein–protein interaction interface are highlighted in red. **C** Co-IP of Nucleolin with RGS6 deletion constructs transfected into human AC-16 cardiomyocytes. **D**–**F** Mice VCM were transduced with RGS6-pEGFP (RGS6 OE) or RGS6 specific shRNA (RGS6 KD) or vector control and harvested 36 hours after transduction. **D** Representative immunoblots and quantification for RGS6, Nucleolin, Nucleophosmin (n = 8). **E** Fold change in Nucleolin and Nucleophosmin mRNA (n = 3). **F** Immunoblots and quantification for Nucleolin pT76 and Nucleolin pT84 (n = 4). β-Actin serves as a loading control for immunoblots. Data were analyzed by one-way ANOVA with Sidak’s post-hoc test. *P < 0.05, **P < 0.01, ***P < 0.001, ****P < 0.0001. ns = not significant. Data are presented as mean ± SEM

**Fig. 2:**
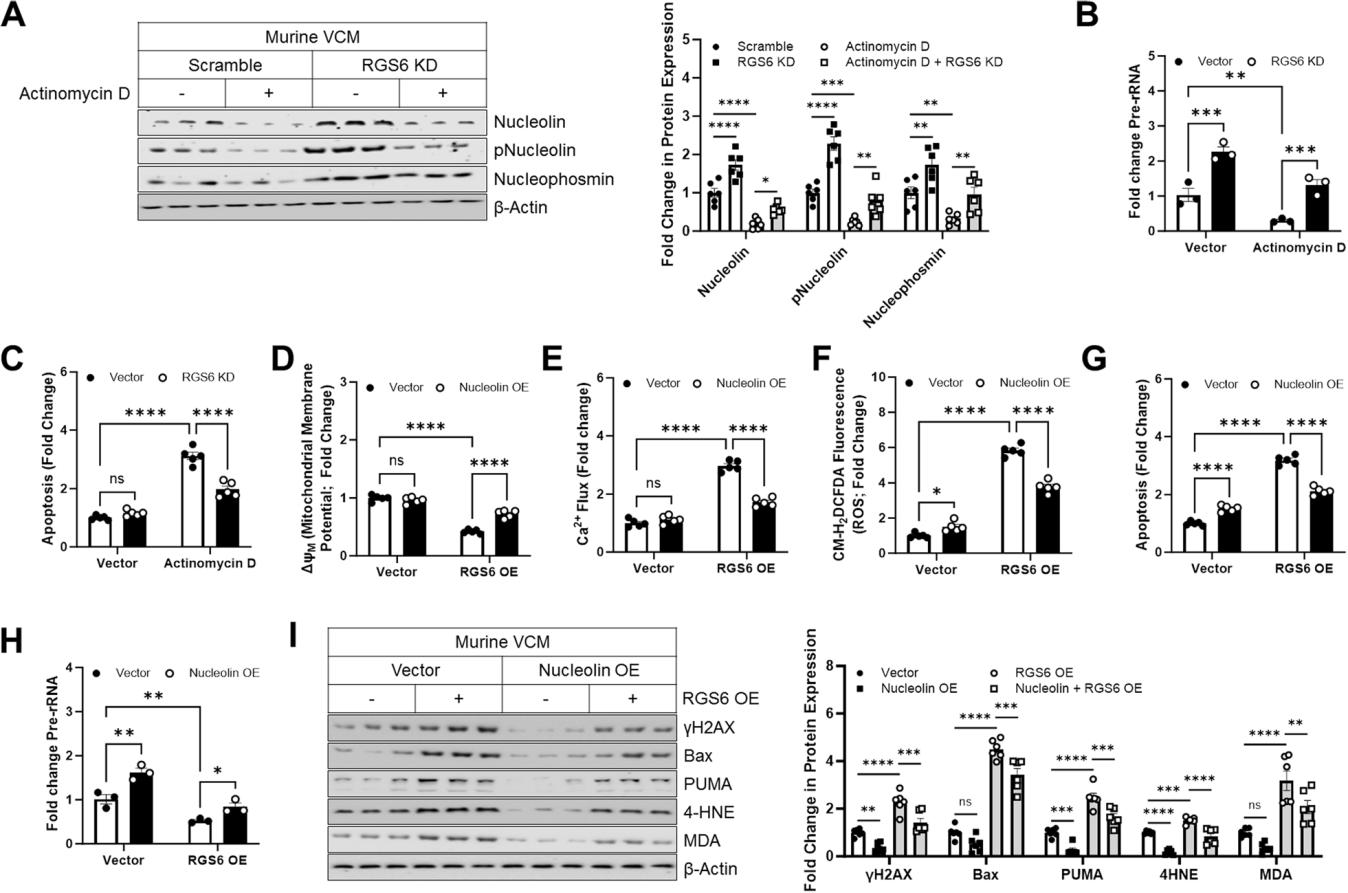
RGS6 promotes nucleolar stress-driven cell death by down-regulating Nucleolin. **A**–**C** Murine VCMs were transduced with scramble or RGS6 specific shRNA ± Actinomycin D (5 µM 16 h). **A** Representative immunoblots with quantification for Nucleolin, pNucleolin (pT76), and Nucleophosmin (n = 6). **B** Fold change in pre-rRNA levels (n = 3). **C** Apoptosis (cytoplasmic histone-associated DNA fragments; n = 5). (D-H) Murine VCMs were transduced with Nucleolin-HA (Nucleolin OE), RGS6-pEGFP (RGS6 OE), both constructs, or vector control and harvested 36 hours after transduction. **D** Mitochondrial membrane potential (ΔΨ_M_; n = 5). **E** Mitochondrial Ca^2+^ Flux (n = 5). **F** CM-H_2_-DCFDA fluorescence (ROS; n = 5). **G** Apoptosis (cytoplasmic histone-associated DNA fragments; n = 5). **H** Fold change in pre-rRNA levels (n = 3). **I** Representative immunoblots and quantification for γH2AX, Bax, PUMA, 4-HNE and MDA.β-Actin served as a loading control for immunoblots. Data were analyzed by two-way ANOVA with Sidak’s post-hoc test. *P < 0.05, **P < 0.01, ***P < 0.001, ****P < 0.0001. ns = not significant. Data are presented as mean ± SEM

RGS6 translocates to the nucleolus in response to several cellular stressors [27], but what function, if any, RGS6 has in this subcellular compartment remained unknown. In fact, RGS6 forms a co-precipitable complex with nucleolar protein Nucleolin, which controls ribosomal synthesis and maturation, in the AC-16 cardiomyocyte cell line (Fig. 1A). MD simulations of a theoretical complex between RGS6 and Nucleolin revealed several amino acids that underwent a significant change in solvent accessibility upon protein–protein binding indicating that these residues form an interaction interface (Fig. 2A, Additional file 1: Table S6). The change in MD simulation variables Rg and RMSD (Additional file 1: Fig. S1B) are also consistent with a stable complex further supported by hydrophobic and ionic interactions particularly between Nucleolin and RGS6 Tyr117 and its adjacent residues (Additional file 1: Fig. S1A). The key RGS6 residues identified by the MD simulation lie within and adjacent to the Dishevelled, Egl-10 and Pleckstrin (DEP) homology domain, previously shown to be required for nucleolar trafficking of RGS6 [26]. Indeed, deletion of the DEP domain but not the Gγ-like (GGL) or catalytic RGS domain decreased the interaction between RGS6 and Nucleolin confirming the veracity of our in silico analysis (Fig. 1C).

Next, we wished to establish the impact of RGS6 on Nucleolin expression and activity. Overexpression of RGS6 in murine VCM (Fig. 1D), human iPSC-derived cardiomyocytes (Additional file 1: Fig. S2A), or AC-16 cells (Additional file 1: Fig. S2C) resulted in decreased Nucleolin expression while RGS6 depletion triggered Nucleolin up-regulation. Nucleolin activity is modified at the transcriptional, and post-translational level [43, 44], and altering RGS6 levels triggered corresponding changes in Nucleolin mRNA levels in mouse and human myocytes (Fig. 1E, Additional file 1: Fig. S2D). Similarly, Nucleolin phosphorylation at two threonine residues (T76 and T84) known to be involved in Nucleolin’s cytoprotective actions [19] was increased following RGS6 knockdown in VCM (Fig. 1F, Additional file 1: Figs. S2B, E).

### Nucleolar stress-induced cell death requires RGS6

Nucleolar stress is a well characterized consequence of the cancer chemotherapeutic Actinomycin D, which intercalates into rDNA and stalls rRNA synthesis by blocking RNA Polymerase I. Actinomycin treatment leads to rapid depletion of Nucleolin, phospho-Nucleolin (T76), and Nucleophosmin in murine VCM (Fig. 2A) and AC-16 cells (Additional file 1: Fig. S3A). Notably, RGS6 knockdown partially mitigated the impact of Actinomycin D on nucleolar protein expression (Fig. 2A, Additional file 1: Fig. S3A). RGS6 depletion was also sufficient to increase synthesis of pre-rRNA, a critical step in ribosome biogenesis that occurs in the nucleolus [45], and prevent Actinomycin D-driven suppression of pre-rRNA generation (Fig. 2B, Additional file 1: Fig. S3B). Finally, while Actinomycin D treatment triggered apoptosis in mouse (Fig. 2C) and human (Additional file 1: Fig. S3C) myocytes, introduction of RGS6 shRNA decreased cell death by approximately 50% (Fig. 2D, Additional file 1: Fig. S3D). These data demonstrate that RGS6 is necessary for myocytes to initiate apoptosis following nucleolar stress.

Next, we wished to establish if RGS6 up-regulation was sufficient to promote nucleolar stress in cardiomyocytes. Indeed, overexpression of RGS6 in murine VCM triggered the intrinsic mitochondrial apoptosis pathway as indicated by loss of mitochondrial membrane potential (ΔΨ_M_) (Fig. 2D), increased mitochondrial calcium flux (Fig. 2E), oxidative stress (Fig. 2F), and an increase in cytoplasmic histone-associated DNA fragments characteristic of cell death (Fig. 2G). These functional readouts were accompanied by increased expression of DNA damage indicator γH2AX, pro-apoptotic proteins Bax and PUMA, and markers of lipid peroxidation and oxidative stress 4-Hydroxynonenal (4-HNE) and malondialdehyde (MDA) (Fig. 2I). RGS6 overexpression also decreased pre-rRNA levels, and, importantly, the impact of RGS6 on nucleolar stress and apoptotic signaling in VCM could be counteracted by restoring Nucleolin expression (Fig. 2D–I) indicating that RGS6-mediated Nucleolin regulation is critical for the pro-apoptotic actions of RGS6 in myocytes. Virtually identical results were obtained in AC-16 cells (Additional file 1: Fig. S3D–G) demonstrating that regulation of cell death pathways by the RGS6/Nucleolin complex is conserved across species.

### RGS6 promotes apoptosis in myocytes by decreasing miRNA-21

**Fig. 3:**
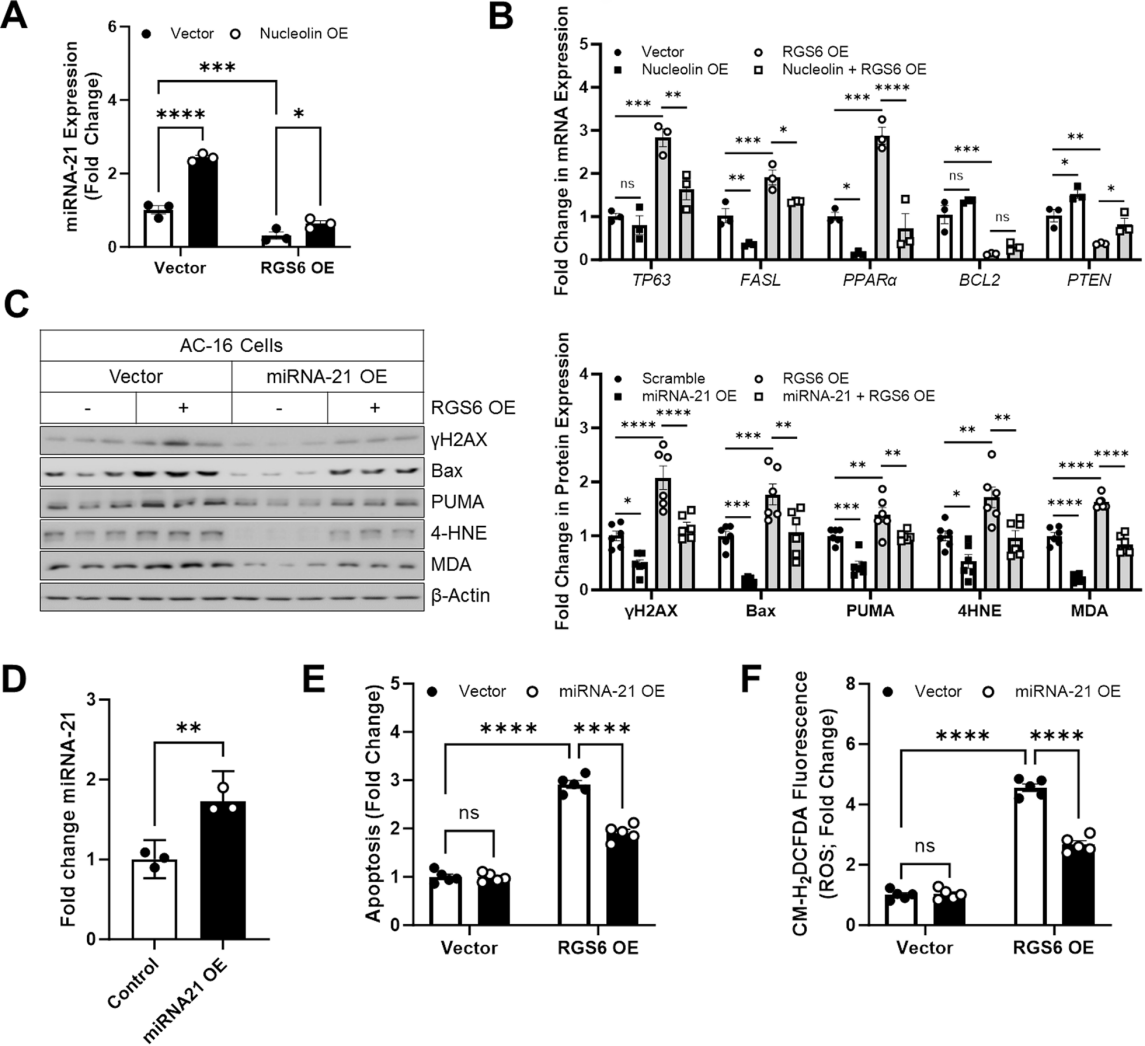
RGS6 controls expression of miRNA-21 via a Nucleolin-dependent mechanism. **A**, **B** AC-16 cells were transduced with Nucleolin-HA (Nucleolin OE), RGS6-pEGFP (RGS6 OE), both constructs, or vector control and harvested 36 hours after transduction. **A** miRNA-21 expression levels (n = 3). **B** mRNA levels of miRNA-21 target genes *TP63*, *FASL*, *PPARα*, *BCL2*, *PTEN* (n = 3). **C**–**F** AC-16 cells were transduced with miRNA-21 (miRNA-21 OE), RGS6-pEGFP (RGS6 OE), both constructs, or vector control and harvested after 36 h). **C** Representative immunoblots with quantification for γH2AX, Bax, PUMA, 4HNE and MDA. β-Actin served as a loading control for immunoblots. **D** Validation of miRNA-21 overexpression (n = 3). **E** Apoptosis (cytoplasmic histone-associated DNA fragments; n = 5). **F** CM-H_2_-DCFDA fluorescence (ROS; n = 5). Data were analyzed by two-way ANOVA with Sidak’s post-hoc test. *P < 0.05, **P < 0.01, ***P < 0.001, ****P < 0.0001. ns = not significant. Data are presented 0.001, ± SEM

Prior work demonstrated that the anti-apoptotic actions of Nucleolin can be attributed to its ability to induce miRNA-21 [16, 18, 19]. Given that RGS6 controls Nucleolin expression, we hypothesized that RGS6 might function to suppress the accumulation of miRNA-21 in myocytes thereby promoting cell death. Indeed, RGS6 overexpression in murine VCM (Fig. 3A) or AC-16 cells (Additional file 1: Fig. S4A) resulted in decreased miRNA-21 levels and counteracted the induction of miRNA-21 resulting from overexpression of Nucleolin (Fig. 3A, Additional file 1: Fig. S4A). RGS6 overexpression also altered expression of known miRNA-21 target genes leading to increased levels of *TP63*, *FASL*, and *PPARα* mRNA as well as decreased *BCL2* and *PTEN* mRNA (Fig. 3B, Additional file 1: Fig. S4B–F). Expression of miRNA-21 target genes following RGS6 overexpression was largely restored by buffering Nucleolin expression with the notable exception of BCL2 mRNA (Fig. 3B, Additional file 1: Fig. S4B–F). In support of a critical role for miRNA-21 suppression in the cytotoxic impacts of RGS6, RGS6-dependent apoptotic signaling (Fig. 3C, E) and oxidative stress (Fig. 3C, F) could be mitigated via overexpression of miRNA-21 (Fig. 3D).

### RGS6 regulates Nucleolin/miRNA-21 in vivo

**Fig. 4:**
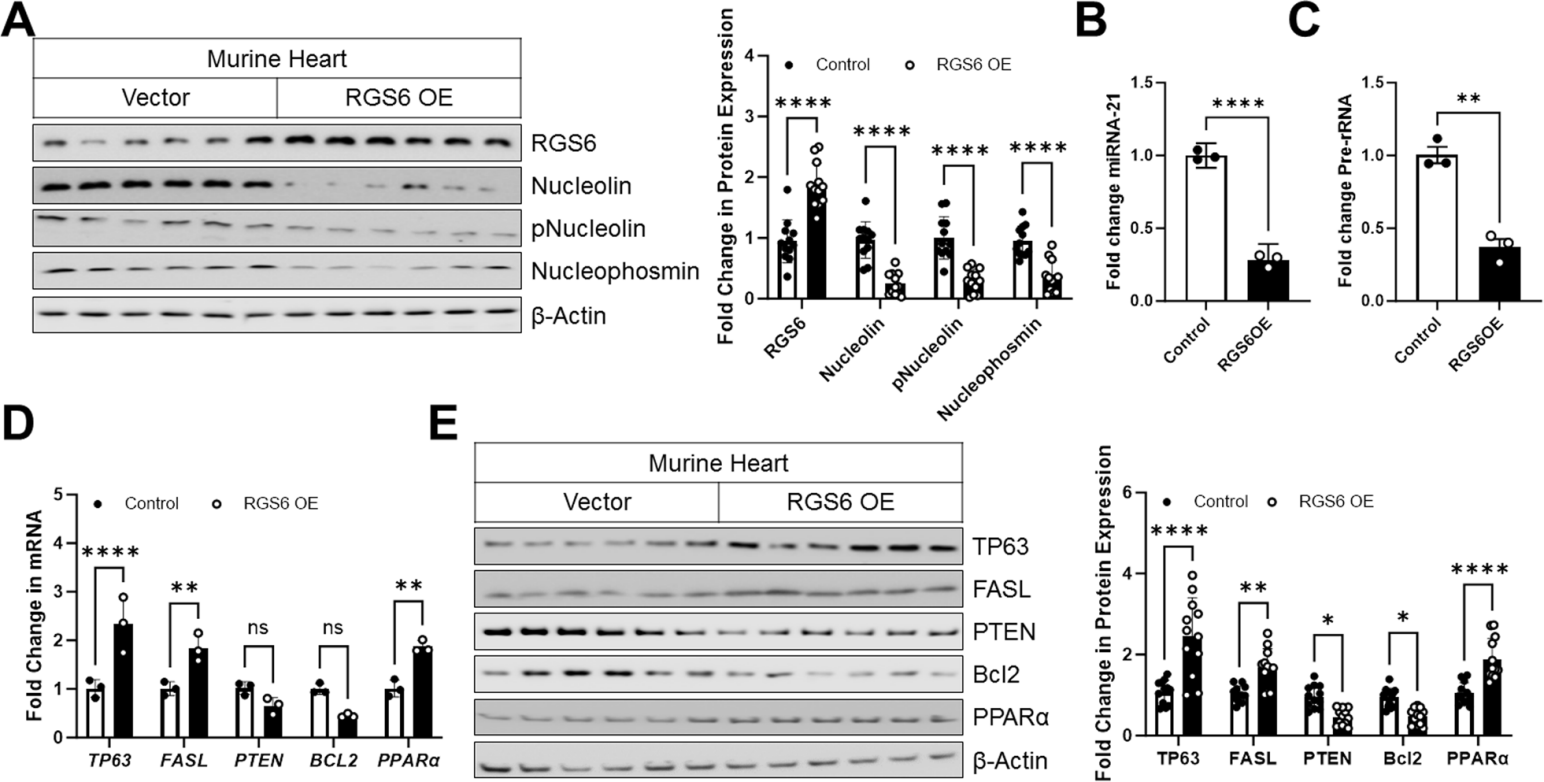
RGS6 controls expression of Nucleolin and miRNA-21 in vivo. An RGS6 encoding viral construct (RGS6 OE) or vector control was introduced into the myocardium of mice aged 1-week. After allowing the virus to be expressed for 10–12 days, animals were sacrificed, and tissues collected for downstream analyses. **A** Representative immunoblots with quantification for RGS6, Nucleolin, pNucleolin (pT76), and Nucleophosmin, (n = 12). **B** miRNA-21 expression (n = 3). **C** Fold change inpre-rRNA(n = 3). **D** mRNA expression of miRNA-21targets*TP63*, *FASL*, *PTEN*, *BCL2*, and *PPARα* (n = 3). **E** Representative immunoblots with quantification for miRNA-21 targets TP63, FASL, PTEN, Bcl-2, and PPARα, (n = 12). β-Actin served as a loading control for immunoblots. Data were analyzed by two-tailed Student’s t-test. *P < 0.05, **P < 0.01, ***P < 0.001, ****P < 0.0001. ns = not significant. Data are presented as mean ± SEM

Having established that RGS6 regulates Nucleolin and miRNA-21 expression in vitro, we next wished to establish that these same impacts could be detected in an intact heart. To this end, we introduced a viral construct encoding RGS6 to the heart of mice via intracardiac injection and confirmed that RGS6 overexpression in the myocardium led to decreased levels of Nucleolin, phospho-Nucleolin, and Nucleophosmin (Fig. 4A) that were accompanied by decrease miRNA-21 expression (Fig. 4B), suppression of pre-rRNA synthesis (Fig. 4C), and altered mRNA (Fig. 4D) and/or protein (Fig. 4E) levels of miRNA-21 targets.

### Doxorubicin alters nucleolar protein expression and triggers nucleolar stress via a RGS6-dependent mechanism

**Fig. 5:**
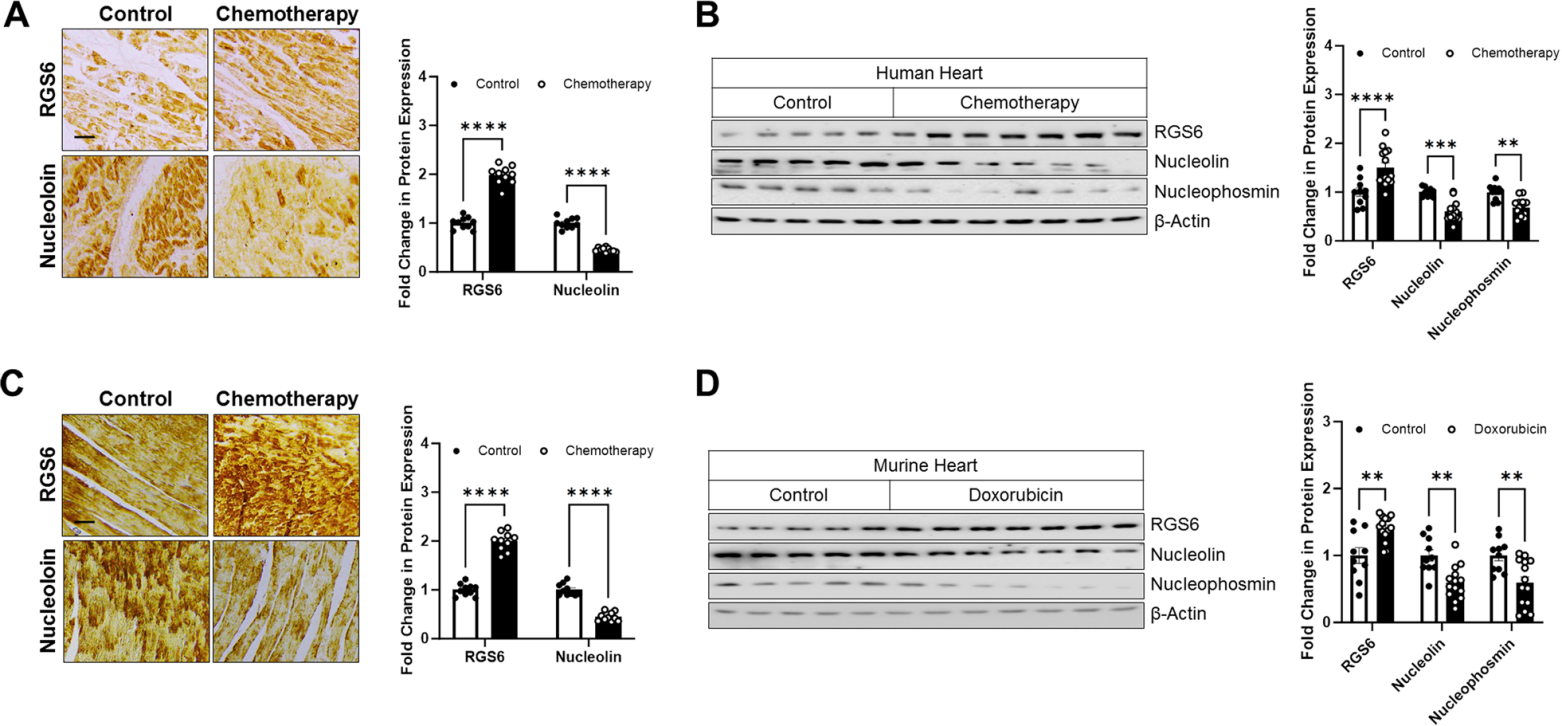
Chemotherapy exposure is associated with parallel changes in RGS6 and Nucleolin/Nucleophosmin expression in heart. **A** Representative staining and quantification of cardiac RGS6 and Nucleolin expression in control (n = 10) or patients with a history of chemotherapy (n = 10) [scale bar = 100 μm]. **B** Representative immunoblots and quantification for RGS6, Nucleolin and Nucleophosmin in the heart of control patients (n = 10) or those with a history of chemotherapy exposure (n = 14). **C**, **D** Mice were treated with saline control (n = 10) or doxorubicin(cumulative dose of 45 mg/kg 9 mg/kg, i.p. every other week) for 10 weeks (**C**) (n = 10), (**D**) (n = 14). Samples were collected 1 week after the last dose for biochemical and histological analyses. (C)Representative staining and quantification of cardiac RGS6 and Nucleolin expression [scale bar = 100 μm]. **D** Representative immunoblots and quantification for RGS6, Nucleolin, and Nucleophosmin (n = 14). β-Actin served as a loading control for immunoblots. Data were analyzed by two-tailed Student’s t-test. *P < 0.05, **P < 0.01, ***P < 0.001, ****P < 0.0001. ns = not significant. Data are presented as mean ± SEM

Given prior work implicating RGS6 in doxorubicin-dependent cardiotoxicity [23], we hypothesized that the ability of doxorubicin to induce RGS6 up-regulation might have a secondary consequence on Nucleolin expression and activity. Indeed, we noted parallel RGS6 induction and Nucleolin depletion in the hearts of human patients with a prior history of chemotherapy exposure via either histochemical (Fig. 5A) or biochemical (Fig. 5B) approaches. Levels of Nucleophosmin were also lower in the chemotherapy-exposed patient group as compared to matched controls (Fig. 5B). Further, chronic, low dose doxorubicin treatment in mice led to similar alterations in expression of RGS6, Nucleolin, and Nucleophosmin (Fig. 5C, D). Importantly, in both mouse and human heart samples, chemotherapy exposure led to alterations in protein expression of miRNA-21 targets TP63, PPARα, FASL, Bcl-2, and PTEN (Additional file 1: Fig. S5A, B). These data led us to postulate that the mechanism we identified linking RGS6 to Nucleolin/miRNA-21 action might play a role in doxorubicin-driven myocyte damage.

**Fig. 6:**
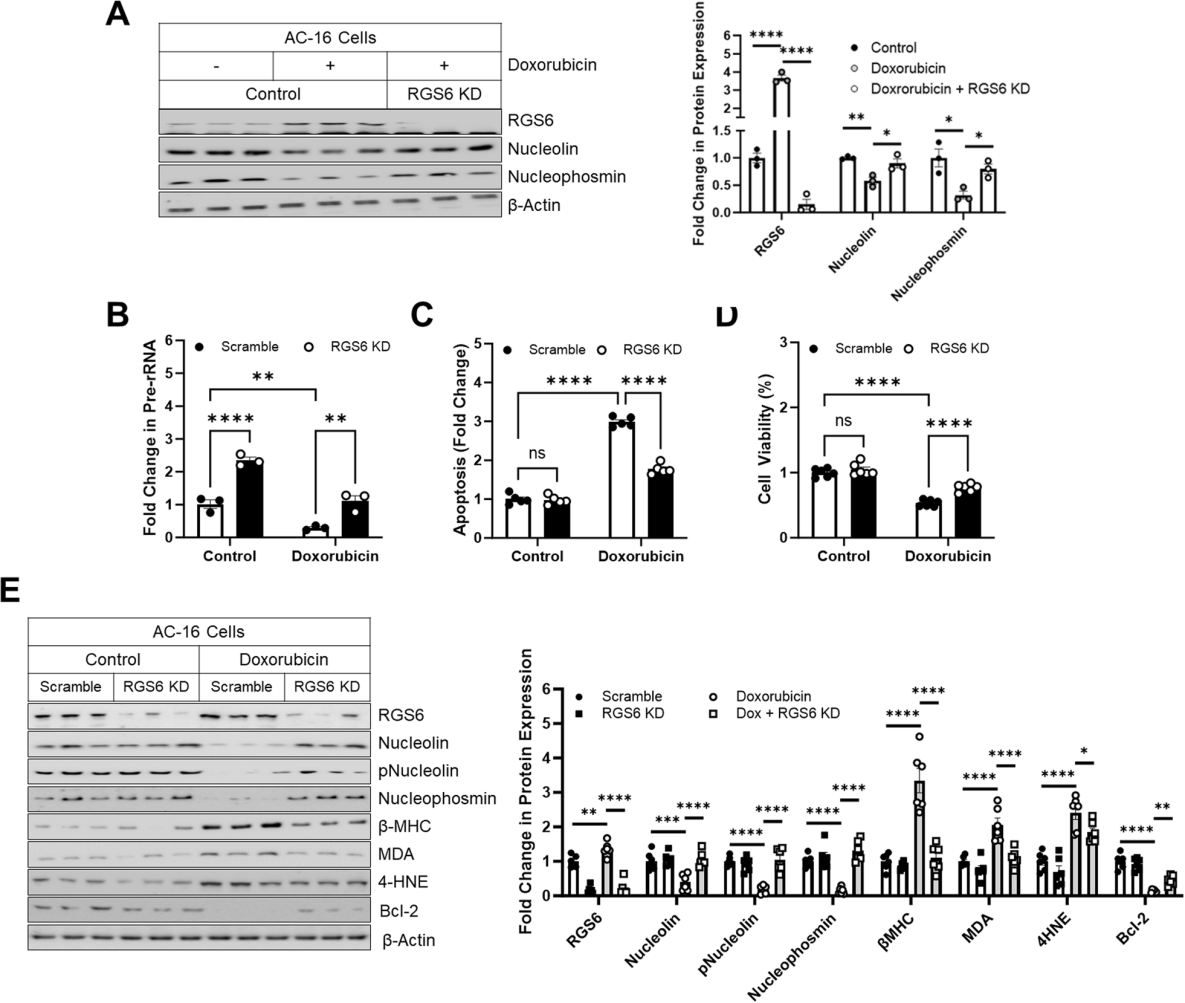
Doxorubicin-dependent nucleolar stress requires RGS6. **A** immunoblots for RGS6, Nucleolin, and Nucleophosmin with quantification (n = 3), in Control or RGS6 KD AC-16 cells ± doxorubicin (3 µM, 16 h). **B**–**E** AC-16 cells were transduced with RGS6 specific shRNA (RGS6 KD) or scramble shRNA ± doxorubicin (3 μM, 16 h). **B** Fold change in pre-rRNA (n = 3). **C** Apoptosis (cytoplasmic histone-associated DNA fragments; n = 5). **D** MTT assay to determine cell viability (n = 6). **E** Immunoblots with quantification for RGS6, Nucleolin, pNucleolin (pT76), Nucleophosmin, β-MHC, MDA, 4HNE, and Bcl-2 (n = 6). β-Actin served as a loading control for immunoblots. Data were analyzed by two-way ANOVA with Sidak’s post-hoc test. *P < 0.05, **P < 0.01, ***P < 0.001, ****P < 0.0001. ns = not significant. Data are presented as mean ± SEM

Indeed, we noted that knockdown of RGS6 from AC-16 cells completed precluded doxorubicin-dependent Nucleolin and Nucleophosmin down-regulation (Fig. 6A). Similarly, knockdown of RGS6 in either AC-16 cells or murine VCM restored pre-rRNA synthesis following doxorubicin exposure (Fig. 6B, Additional file 1: Fig. S6A) with a corresponding decrease in apoptosis (Fig. 6C, Additional file 1: Fig. S6B) and increase in cell viability (Fig. 6D, Additional file 1: Fig. S6C). Consistent with a critical role for RGS6 in doxorubicin-driven myocyte toxicity, the ability of doxorubicin to alter expression of nucleolar proteins Nucleolin, phospho-Nucleolin, Nucleophosmin; hypertrophy-associated protein β myosin heavy chain (β-MHC); oxidative stress markers MDA and 4-HNE; and anti-apoptotic protein Bcl-2 was mitigated following RGS6 knockdown (Fig. 6E, Additional file 1: Fig. S6D).

### Nucleolin depletion or miRNA-21 inhibition phenocopies the cytotoxic impact of doxorubicin in myocytes

**Fig. 7:**
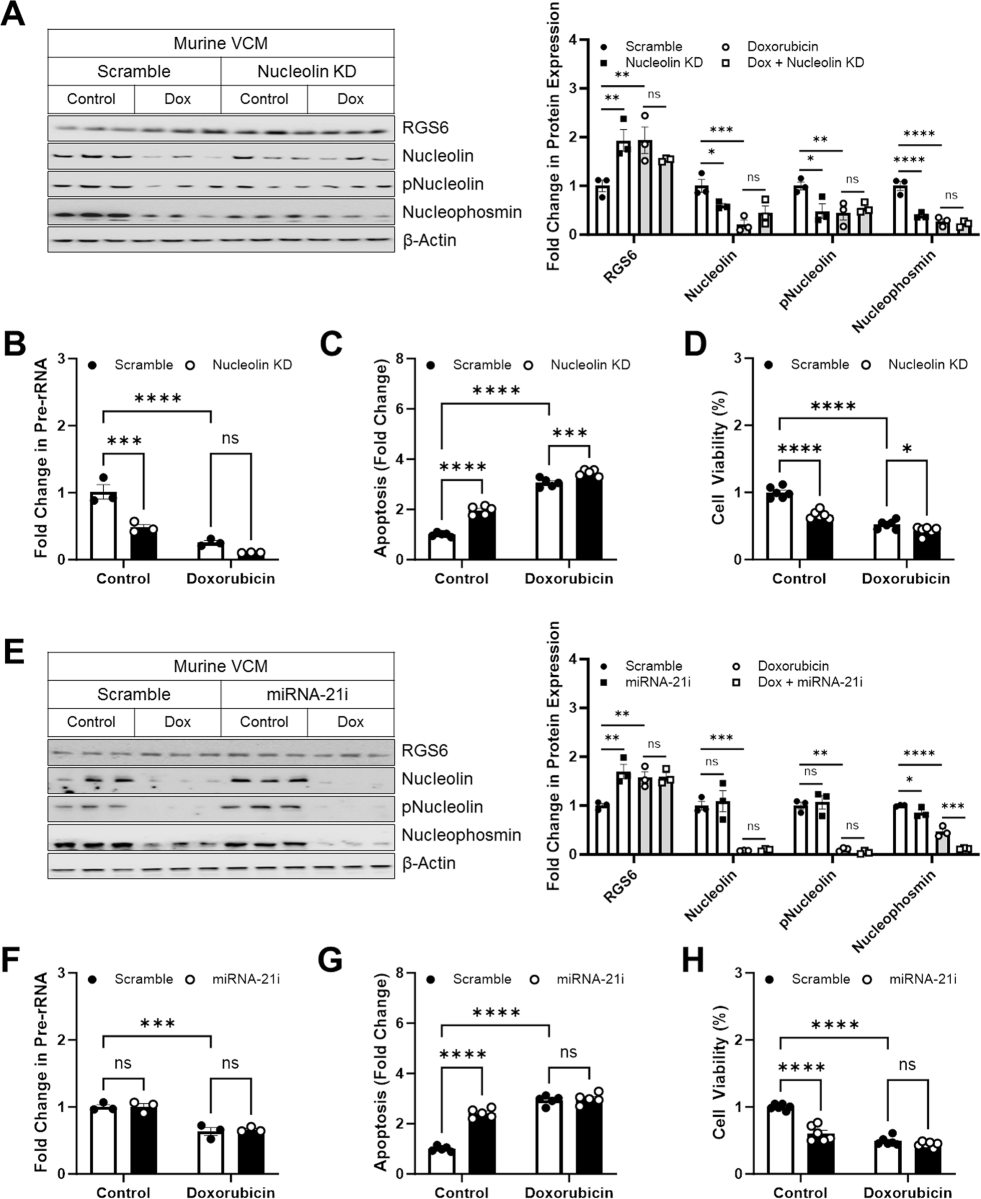
Inhibition of miRNA-21 or Nucleolin depletion phenocopies the impact of RGS6 on nucleolar stress. **A**–**D** Murine VCMs were transduced with scramble or Nucleolin specific shRNA (Nucleolin KD) ± doxorubicin (3 μM, 16 h). **A** Representative immunoblots and quantification for RGS6, Nucleolin, pNucleolin (pT76), and Nucleophosmin (n = 3). **B** Fold change in pre-rRNA (n = 3). **C** Apoptosis (cytoplasmic histone-associated DNA fragments; n = 5). **D** MTT assay to determine cell viability (n = 6). **E**–**H** Murine VCMs were transduced with scramble or miRNA-21 inhibitor (miRNA-21i) ± doxorubicin (3 μM, 16 h). **E** Immunoblots and quantification for RGS6, Nucleolin, pNucleolin (pT76), and Nucleophosmin (n = 3). **F** Fold change in pre-rRNA (n = 3). **G** Apoptosis (cytoplasmic histone-associated DNA fragments; n = 5). **H** MTT assay to determine cell viability (n = 6). β-Actin served as the loading control for the immunoblots. Data were analyzed by two-way ANOVA with Sidak’s post-hoc test. *P < 0.05, **P < 0.01, ***P < 0.001, ****P < 0.0001. ns = not significant. Data are presented as mean ± SEM

As we noted a consistent impact of doxorubicin on expression of Nucleolin and RGS6 across species and preparations, we hypothesized that RGS6 up-regulation and Nucleolin depletion might represent a critical mechanism whereby chemotherapy exposure triggers myocyte dysfunction and death. If true, knockdown of Nucleolin in VCM should be sufficient to induce cell death and/or sensitize cells to doxorubicin-driven apoptosis. Introduction of shRNA targeting Nucleolin successfully decreased Nucleolin and phospho-Nucleolin expression in murine VCM (Fig. 7A) or human AC-16 cells (Additional file 1: Fig. S7A) to levels similar to those in doxorubicin treated cells. Intriguingly, Nucleolin depletion also led to RGS6 up-regulation indicating these two proteins participate in negative reciprocal regulation of the others’ stability or production (Fig. 7A, Additional file 1: Fig. S7A). As expected, Nucleolin knockdown decreased production of pre-rRNA and failed to further decrease pre-rRNA levels in doxorubicin treated cells (Fig. 7B, Additional file 1: Fig. S7B). Introduction of Nucleolin shRNA also increased apoptosis (Fig. 7C, Additional file 1: Fig. S7C) and decreased cell viability (Fig. 7D, Additional file 1: Fig. S7D) in myocytes with a small, but significant potentiation of cell loss in myocytes treated with doxorubicin and Nucleolin shRNA (Fig. 7C, D, Additional file 1: Fig. S7C–D).

Given prior work identifying miRNA-21 as the key mediator of the anti-apoptotic actions of Nucleolin [16, 18, 19] we expected inhibition of miRNA-21 to also lead to death of myocytes while possibly bypassing the necessity for nucleolar stress. Indeed, introduction of an inhibitor of miRNA-21 had only modest impacts on Nucleophosmin expression and failed to alter expression of Nucleolin or phospho-Nucleolin in control or doxorubicin-treated VCM (Fig. 7E) or AC-16 cells (Additional file 1: Fig. S7E). However, miRNA-21 blockade did increase expression of RGS6 (Fig. 7E, Additional file 1: Fig. S7E) indicating RGS6 may be a miRNA-21 target gene and that Nucleolin may alter RGS6 expression via a miRNA-21-dependent mechanism. Finally, while the addition of miRNA-21 failed to alter pre-rRNA production (Fig. 7F, Additional file 1: Fig. S7F), it did increase apoptosis (Fig. 7G, Additional file 1: Fig. S7G) and decrease viable myocytes (Fig. 7H, Additional file 1: Fig. S7H) without potentiating the cytotoxic impact of doxorubicin. Together these data point to a critical role for Nucleolin and miRNA-21 in alleviation of doxorubicin-driven cell death in myocytes.

### RGS6 knockdown prevents doxorubicin-dependent disruption in Nucleolin/miRNA-21 activity

**Fig. 8:**
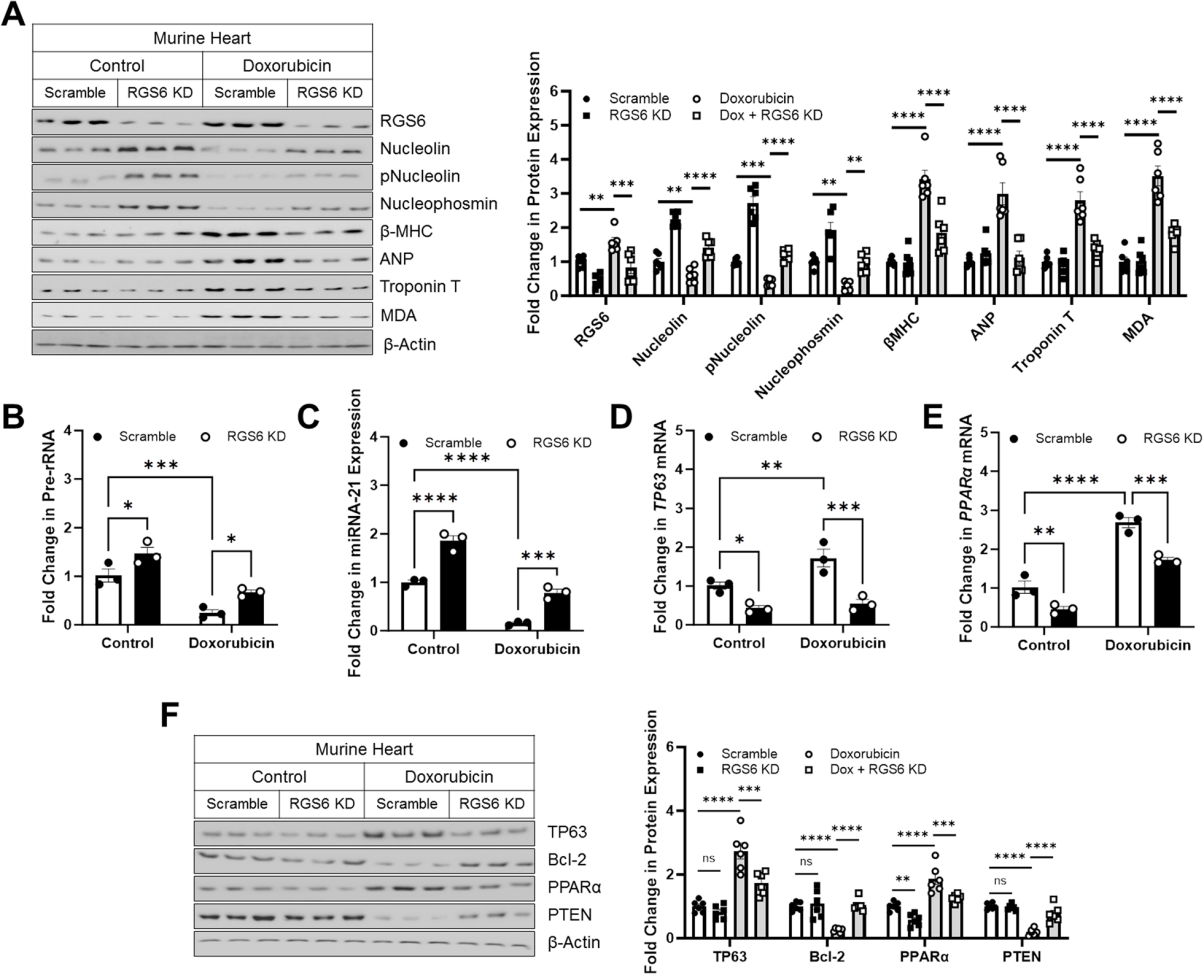
RGS6 knockdown in heart prevents doxorubicin-dependent changes in Nucleolin and miRNA-21 expression and activity. Intracardiac injections of scramble or RGS6 specific shRNA (RGS6 KD) were administered to mice aged 1-week and followed by treatment with doxorubicin (cumulative dose of 45 mg/kg 9 mg/kg, i.p. every other week) or saline for 10 weeks beginning at age 8–10 weeks. Samples were collected 1 week after the last dose later for biochemical and histological analyses. **A** Representative immunoblots with quantification for RGS6, Nucleolin, pNucleolin (pT76), Nucleophosmin, β-MHC, ANP, Troponin T, and MDA (n = 6). **B** Fold change inpre-rRNA, (n = 3). **C** miRNA-21expression (n = 3). Expression of miRNA-21 target genes **D** *TP63* and **E** *PPARα* (n = 3). **F** Representative immunoblots for miRNA-21 targets TP63, Bcl-2, PPARα, and PTEN (n = 6). β-Actin served as the loading control for the immunoblots. Data were analyzed by two-way ANOVA with Sidak’s post-hoc test. *P < 0.05, **P < 0.01, ***P < 0.001, ****P < 0.0001. ns = not significant. Data are presented as mean ± SEM

Finally, we wished to establish if RGS6 is required to promote disruptions in Nucleolin/miRNA-21 activity in vivo. To this end, we introduced RGS6 shRNA directly to the murine myocardium in control animals or those exposed to a chronic, low dose doxorubicin treatment paradigm. As we observed in isolated myocytes, RGS6 knockdown prevented doxorubicin-driven loss of Nucleolin, phospho-Nucleolin, and Nucleophosmin expression in the heart (Fig. 8A). In fact, RGS6 shRNA alone was sufficient to induce robust up-regulation in Nucleolin and Nucleophosmin levels in heart tissue (Fig. 8A) emphasizing, again, that RGS6 is responsible for controlling Nucleolin expression in the myocardium. Doxorubicin also increased expression of hypertrophy and heart failure markers β-MHC, atrial natriuretic peptide (ANP), and troponin T as well as the oxidative stress marker MDA via a RGS6-dependent mechanism (Fig. 8A). Consistent with the impact of RGS6 on Nucleolin levels, we noted that RGS6 KD led to increase pre-rRNA synthesis (Fig. 8B) and miRNA-21 expression (Fig. 8C) and counteracted the impact of doxorubicin on these measures. Similarly, mRNA levels of miRNA-21 targets *TP63* (Fig. 8D) and *PPARα* (Fig. 8E) decreased following RGS6 KD at baseline and following doxorubicin exposure. Changes in mRNA expression were largely reflected at the protein level where, once again, RGS6 depletion prevented the ability of doxorubicin to alter levels of pro-(TP63, PPARα) and anti-apoptotic (Bcl-2, PTEN) proteins (Fig. 8F). These data are consistent with a model wherein doxorubicin induces expression of RGS6 which promotes apoptosis by suppressing the actions of Nucleolin/miRNA-21.

## Discussion

Though the canonical functions of RGS proteins in GPCR regulation would require their presence in the cytosol or at the cell membrane, we have known for decades that members of this diverse protein family localize to other subcellular compartments. For example, members of the R7 subfamily, which includes RGS6, 7, 9 and 11, have been shown to shuttle between the plasma membrane and nucleus, a mechanism believed to modulate their GPCR regulatory capacity by sequestering R7 family members away from active G proteins [46–49]. Here, we provide evidence for a completely novel role for RGS6 in nucleolar function where the ability of RGS6 to bind to and suppress the expression and activity of nucleolar protein Nucleolin. RGS6 is both necessary and sufficient to alter Nucleolin levels following nucleolar stress and plays a key role in counteracting the anti-apoptotic actions of Nucleolin in response to genotoxic stressors such as the chemotherapeutic drug doxorubicin. Though several mechanisms have been proposed whereby RGS6 up-regulation could lead to cell death including via direct modulation of the DNA damage signaling [24, 25] and intrinsic mitochondrial apoptosis pathways [22], our observation that overexpression of Nucleolin or Nucleolin effector miRNA-21 mitigates the detrimental impact of RGS6 on mitochondrial function, oxidative stress, and cell death lends credence to the idea that RGS6-dependent regulation of Nucleolin/miRNA-21 is required for its pro-apoptotic actions in myocytes. Importantly, RGS6 depletion in the murine myocardium largely prevented doxorubicin-driven Nucleolin/miRNA-21 down-regulation, blockade of nucleolar output, and alterations in miRNA-21 target gene expression identifying RGS6 as a key regulator of nucleolar signaling in vivo. Thus, we propose a model whereby RGS6 promotes death of cardiomyocytes by preventing Nucleolin-dependent production of miRNA-21 which would otherwise target the mRNA of several pro-apoptotic genes for degradation. Given that loss of functional myocytes and the resultant pathogenic remodeling represent a primary contributor to eventual heart failure, our data point to a key role for RGS6 in the pathogenesis of heart disease, which remains the leading cause of death in both men and women worldwide. Indeed, prior work has demonstrated that RGS6 null mice are largely protected against doxorubicin-dependent cardiac injury [23].

In neurons, reversible palmitoylation of the membrane anchor R7 binding protein (R7BP) occludes two nuclear localization sequences and controls targeting of R7 family RGS proteins to the plasma membrane [46–49]. However, R7BP expression is restricted to the nervous system and no expression has been detected in non-neuronal cell types [50]. Thus, whereas very little expression of R7 family members is detectable in the nucleus of neurons in the absence of stimulation [51], the same may not be true in other cell types including cardiomyocytes. This may also explain why investigations into non-canonical functions of R7 family RGS6 proteins have primarily been confined to peripheral organ systems where RGS6 has been shown to directly interact with several nuclear proteins including ATM [25], DNMT1 via DNMT1 associated protein DMAP1 [26, 52], the histone acetyltransferase Tip60 [52], and now Nucleolin. Intriguingly, initiation of the DNA damage response and ATM activation triggers nucleolar reorganization [28], which in turn leads to stabilization of p53 and facilitates apoptosis [53]. Cells lacking DNMT1 also show altered epigenetic regulation of rDNA genes indicating DNMT1 localizes to the nucleolus and controls ribosome biogenesis [54]. Coincidently, Tip60 also localizes to the nucleolus [55] and is present at sides of active rDNA transcription [56]. These data imply that, by scaffolding a diverse array of nucleolar proteins, RGS6 may act as a master regulator of nucleolar output. In support of this theory, each of these interacting partners bind to a distinct portion of the RGS6 protein with Tip60 and ATM binding in the RGS domain [25, 52], DMAP1/DNMT1 binding to the GGL domain [26], and Nucleolin binding the DEP domain. RGS6, then, might function to localize these partners closer in proximity to each other or a yet-to-be identified target.

The lack of tight complex formation with R7BP, which binds to R7 family members via the DEP domain [50], in cardiomyocytes likely facilitates the RGS6-Nucleolin interaction. However, the exact means by which RGS6 modulates Nucleolin expression and activity remains unclear. Our data show a clear diminution in Nucleolin expression with increased RGS6 levels and a corresponding Nucleolin up-regulation following RGS6 depletion across species and in both cardiac tissue and isolated myocytes. This is accompanied by corresponding changes in Nucleolin mRNA indicating RGS6 might control Nucleolin transcription. Nucleolin phosphorylation at T76 and T84 is required for its ability to protect against apoptosis and regulate miRNA-21 in myocytes [20], and RGS6 also modulates Nucleolin phosphorylation levels, though this may just be a secondary consequence of changes in the total pool of Nucleolin. One intriguing possibility is that, like R7 family member RGS7, which bidirectionally controls acetylation of p65 [57], RGS6/Tip60 might direct Nucleolin acetylation [58], which has been shown to influence proteasomal Nucleolin degradation [59]. We should also note that Nucleolin knockdown alone was sufficient to trigger RGS6 up-regulation indicating that Nucleolin and RGS6 directly antagonize the activity of the other. As miRNA-21 inhibition resulted in the same RGS6 induction without influencing Nucleolin levels, it is likely that RGS6 mRNA is directly degraded by miRNA-21 or that RGS6 transcription or translation is controlled by a miRNA-21 target. RGS6 expression can be induced by several cytotoxic stimuli including alcohol [60], hyperlipidemia [25], and chemotherapeutics [23], an effect that may be achieved or amplified by down-regulation of miRNA-21.

Three R7 family RGS proteins are expressed in heart, RGS6, RGS7 and RGS11, all of which compete for a shared pool of their co-stabilizing binding partner G protein β 5 (Gβ_5_) [23, 29, 30]. Intriguingly, doxorubicin treatment shifts expression of all 4 proteins in heart causing an increase in RGS6, RGS7 and Gβ_5_ but a decrease in RGS11 [23, 29, 30, 33]. Of the three R7 family members present in the myocardium, RGS6 and RGS7 share the greatest homology (75%), but, nevertheless, appear to have unique functions. Both proteins are profoundly proapoptotic in cardiomyocytes [23, 29] but, while RGS6 has been implicated in DNA damage signaling via interactions with ATM [23, 25] and now in nucleolar stress signaling, RGS7 promotes mitochondrial dysfunction and cell death in cardiomyocytes by forming a complex with Ca^2+^/calmodulin-dependent protein kinase II (CAMKII) [29]. RGS7 has also been implicated in cardiac inflammation via its ability to bidirectionally modulate acetylation of the p65 subunit of the nuclear factor κ B (NF-κB) complex [56]. RGS11 directly antagonizes the pro-fibrotic and pro-apoptotic actions of RGS7 [30, 61], but, as Gβ_5_ knockdown protects the heart against doxorubicin-driven damage [33], it is likely that RGS7 and RGS6 activity predominate in the presence of cytotoxic stress. These distinct and sometimes diametrically opposed actions of R7 family members have important implications in the clinical context as, while inhibition of either RGS6 or RGS7 may be cardioprotective, RGS6, but not RGS7 is required for the cancer killing actions of doxorubicin [24, 29] and functions as a tumor suppressor in bladder and breast cancers [62, 63]. RGS6 is also the primary G protein activating protein (GAP) for M2 muscarinic receptors in pacemaking portions of the heart where it functions as a break on parasympathetic drive [64, 65] and, as a result, is associated with heart rate variability in humans [66] and mice [67]. Thus, while a non-targeted therapeutic aimed at RGS6 inhibition might be cardioprotective, it might also compromise the therapeutic efficacy of chemotherapy, drive de novo carcinogenesis, or alter cardiac conduction. It is possible, however, that by interfering with specific RGS6 effectors, such as Nucleolin, more selective disruption of RGS6’s cardiotoxic actions could be achieved.

Nucleolin levels in heart have been shown to correlate strongly with cardiac function with low Nucleolin protein levels associated with better left ventricular end diastolic and systolic diameters in individuals with ischemic or dilated cardiomyopathy [6]. From these data, one might infer that high Nucleolin levels drive cardiac damage in direct contradiction to the data presented in this work. However, the exact impact of Nucleolin on cardiac outcome likely depends on where Nucleolin is acting and what stage of the cardiac remodeling process is ongoing. For example, Nucleolin is cleaved and inactivated in the early phases of ischemia–reperfusion (I/R) injury [14] and decreased immediately (24 h) following myocardial infarction (MI), but Nucleolin expression is elevated from day 7–28 post-MI [15]. During the later stages of hypertrophy and heart failure, Nucleolin-chromatin binding is also enhanced [68]. In addition to our data demonstrating that Nucleolin depletion compromises the viability of cardiomyocytes, others have observed that Nucleolin protects myocytes against cell death induced by hypoxia or oxidative injury [14, 17] and in models of acute MI and I/R injury, Nucleolin down-regulation is associated with impaired cardiac function [14, 15]. However, Nucleolin also promotes release of pro-inflammatory cytokines and is required for polarization of M2 macrophages, a critical pool of immune cells important for cardiac repair [15, 69]. As RGS6 appears to directly counteract the anti-apoptotic actions of Nucleolin, we would predict that disrupting RGS6-Nucleolin binding might increase myocyte resiliency toward acute cardiotoxic insult without disrupting the cardioprotective actions of Nucleolin. These data add to our understanding of RGS protein action away from the GPCR laden plasma membrane and identify the RGS6/Nucleolin complex as a potential target for therapeutic strategies aimed at preventing loss of functional myocytes. While such an approach would likely have clinical utility as an adjuvant for individuals undergoing chemotherapy and at high risk for cardiac injury, it might also prove effective following ischemic injury or in disease states associated with significant cardiac comorbidities.

## Conclusions

Here we provide new evidence that RGS6 induction following exposure of cardiac myocytes to the chemotherapeutic drug doxorubicin, RGS6 suppresses the activity of nucleolar protein Nucleolin. Consequently, the production of miRNA-21, which suppresses translation of mRNAs encoding key proapoptotic proteins, is decreased. This mechanism represents one way in which RGS6 drives oxidative stress, mitochondrial dysfunction, and death of cardiomyocytes. Thus, decreasing RGS6 expression in the murine myocardium provides marked protection against chemotherapy-dependent myocyte loss, actions that would be expected to help maintain cardiac output and prevent heart failure in patients undergoing chemotherapy particularly those at high risk for adverse cardiac events.

## Limitations and future directions

Future work will seek to more clearly define the dynamic interrelationship between Nucleolin and RGS6 in the heart. We provide one example herein of how RGS6 induction modifies Nucleolin action, but it remains unclear whether RGS6 plays a key role in modulating nucleolar output in the absence of cytotoxic stress or if additional disease states characterized by cardiac injury would result in recruitment of RGS6-dependent Nucleolin suppression. In addition, our in vivo knockdown strategy would decrease, but not eliminate, RGS6 expression in all cell types. We focused heavily on the myocyte intrinsic actions of RGS6, but a critical role of RGS6 in myocyte apoptosis does not preclude a contribution of RGS6 to cellular signaling in resident fibroblasts or immune cells, which play a key role in repair as well as maladaptive fibrosis after loss of functional myocytes. We should also note that Nucleolin is not the only RGS6 binding partner in myocytes, and it remains to be determined how/when/if RGS6 senses cellular stress signals and directs the activity of each effector. Indeed, our observation that restoring expression of Nucleolin or miRNA-21 only partially abrogates the pro-apoptotic actions of RGS6 in myocytes suggests other mechanisms likely contribute. Systematically dissecting the web of signaling cascades modulated by RGS6 in heart will allow for the generation of therapeutics capable of selectively mitigating RGS6-dependent cell death.

### Supplementary Information

**Additional file 1: Figure S1.** Supplemental molecular modeling of the RGS6-Nucleolin complex. **Figure S2.** RSG6 modules expression of Nucleolin and Nucleophosmin in human cardiomyocytes. **Figure S3.** RGS6 promotes nucleolar stress-driven cell death by down-regulating Nucleolin in human myocytes. **Figure S4.** RGS6 controls expression of miRNA-21 and target genes in murine VCM. **Figure S5.** Chemotherapy alters expression of miRNA-21 target genes in human and murine myocardium. **Figure S6.** RGS6 drives changes in nucleolar function in murine VCM. **Figure S7.** Inhibition of miRNA-21 or Nucleolin depletion phenocopies the impact of RGS6 on nucleolar stress in human cardiomyocytes. **Table S1.** Reagent List. **TableS2.** Cell Line List. **Table S3.** Antibody List. **Table S4.** Assay Kit List. **Table S5.** Primer List. **Table S6.** The residues undergoing significant change in the solvent accessible surface area (SASA) upon complex formation between RGS6 and Nucleolin. **Table S7.** Clinical Data for chemotherapy-treated heart autopsy samples: controls (Con) 1-12; chemotherapy patients without detectable fibrosis (C-F) 1-8; and chemotherapy patients with detectable fibrosis (C+F) 1-8.

## Data Availability

All the relevant information about the data is either mentioned in the manuscript or in the supplemental file. All the western blots original were also submitted in the supplemental section.

## References

[1] Moss T, Langlois F, Gagnon-Kugler T, Stefanovsky V (2007). A housekeeper with power of attorney: the rRNA genes in ribosome biogenesis. Cell Mol Life Sci.

[2] Gupta S, Santoro R (2020). Regulation and roles of the nucleolus in embryonic stem cells: from ribosome biogenesis to genome organization. Stem Cell Reports.

[3] Iarovaia OV, Minina EP, Sheval EV, Onichtchouk D, Dokudovskaya S, Razin SV, Vassetzky YS (2019). Nucleolus: a central hub for nuclear functions. Trends Cell Biol.

[4] Yang K, Yang J, Yi J (2018). Nucleolar stress: hallmarks, sensing mechanism and diseases. Cell Stress.

[5] Yan D, Hua L (2022). Nucleolar stress: friend or foe in cardiac function?. Front Cardiovasc Med.

[6] Rosello-Lleti E, Rivera M, Cortes R, Azorin I, Sirera R, Martinez-Dolz L, Hove L, Cinca J, Lago F, Gonzalez-Juanatey JR, Salvador A, Portoles M (2012). Influence of heart failure on nucleolar organization and protein expression in human hearts. Biochem Biophys Res Commun.

[7] Wu J, Li K, Liu Y, Feng A, Liu C, Adu-Amankwaah J, Ji M, Ma Y, Hao Y, Bu H, Sun H (2023). Daidzein ameliorates doxorubicin-induced cardiac injury by inhibiting autophagy and apoptosis in rats. Food Funct.

[8] Ahmad Y, Boisvert FM, Gregor P, Cobley A, Lamond AI (2009). NOPdb: nucleolar proteome database–2008 update. Nucleic Acids Res.

[9] Andersen JS, Lam YW, Leung AK, Ong SE, Lyon CE, Lamond AI, Mann M (2005). Nucleolar proteome dynamics. Nature.

[10] Boisvert FM, Lam YW, Lamont D, Lamond AI (2010). A quantitative proteomics analysis of subcellular proteome localization and changes induced by DNA damage. Mol Cell Proteomics.

[11] Moore HM, Bai B, Boisvert FM, Latonen L, Rantanen V, Simpson JC, Pepperkok R, Lamond AI, Laiho M (2011). Quantitative proteomics and dynamic imaging of the nucleolus reveal distinct responses to UV and ionizing radiation. Mol Cell Proteomics.

[12] Siddiqi S, Gude N, Hosoda T, Muraski J, Rubio M, Emmanuel G, Fransioli J, Vitale S, Parolin C, D'Amario D, Schaefer E, Kajstura J, Leri A, Anversa P, Sussman MA (2008). Myocardial induction of nucleostemin in response to postnatal growth and pathological challenge. Circ Res.

[13] Avitabile D, Bailey B, Cottage CT, Sundararaman B, Joyo A, McGregor M, Gude N, Truffa S, Zarrabi A, Konstandin M, Khan M, Mohsin S, Volkers M, Toko H, Mason M, Cheng Z, Din S, Alvarez R, Fischer K, Sussman MA (2011). Nucleolar stress is an early response to myocardial damage involving nucleolar proteins nucleostemin and nucleophosmin. Proc Natl Acad Sci U S A.

[14] Jiang B, Zhang B, Liang P, Chen G, Zhou B, Lv C, Tu Z, Xiao X (2013). Nucleolin protects the heart from ischaemia-reperfusion injury by up-regulating heat shock protein 32. Cardiovasc Res.

[15] Tang Y, Lin X, Chen C, Tong Z, Sun H, Li Y, Liang P, Jiang B (2021). Nucleolin improves heart function during recovery from myocardial infarction by modulating macrophage polarization. J Cardiovasc Pharmacol Ther.

[16] Sun H, Tong Z, Fang Y, Jiang B, Liang P, Tang Y, Li Y, Wu Y, Xiao X (2018). Nucleolin protects against doxorubicin-induced cardiotoxicity via upregulating microRNA-21. J Cell Physiol.

[17] Jiang B, Zhang B, Liang P, Song J, Deng H, Tu Z, Deng G, Xiao X (2010). Nucleolin/C23 mediates the antiapoptotic effect of heat shock protein 70 during oxidative stress. FEBS.

[18] Lyu QL, Jiang BM, Zhou B, Sun L, Tong ZY, Li YB, Tang YT, Sun H, Liu MD, Xiao XZ (2018). MicroRNA profiling of transgenic mice with myocardial overexpression of nucleolin. Chin Med J (Engl).

[19] Tong Z, Tang Y, Jiang B, Wu Y, Liu Y, Li Y, Xiao X (2019). Phosphorylation of nucleolin is indispensable to upregulate miR-21 and inhibit apoptosis in cardiomyocytes. J Cell Physiol.

[20] Buscaglia LE, Li Y (2011). Apoptosis and the target genes of microRNA-21. Chin J Cancer.

[21] Stewart A, Fisher RA (2015). Introduction: G protein-coupled receptors and RGS proteins. Prog Mol Biol Transl Sci.

[22] Maity B, Yang J, Huang J, Askeland RW, Bera S, Fisher RA (2011). Regulator of G protein signaling 6 (RGS6) induces apoptosis via a mitochondrial-dependent pathway not involving its GTPase-activating protein activity. J Biol Chem.

[23] Yang J, Maity B, Huang J, Gao Z, Stewart A, Weiss RM, Anderson ME, Fisher RA (2013). G-protein inactivator RGS6 mediates myocardial cell apoptosis and cardiomyopathy caused by doxorubicin. Cancer Res.

[24] Huang J, Yang J, Maity B, Mayuzumi D, Fisher RA (2011). Regulator of G protein signaling 6 mediates doxorubicin-induced ATM and p53 activation by a reactive oxygen species-dependent mechanism. Cancer Res.

[25] Mahata T, Sengar AS, Basak M, Das K, Pramanick A, Verma SK, Singh PK, Biswas S, Sarkar S, Saha S, Chatterjee S, Das M, Stewart A, Maity B (2021). Hepatic Regulator of G Protein Signaling 6 (RGS6) drives non-alcoholic fatty liver disease by promoting oxidative stress and ATM-dependent cell death. Redox Biol.

[26] Liu Z, Fisher RA (2004). RGS6 interacts with DMAP1 and DNMT1 and inhibits DMAP1 transcriptional repressor activity. J Biol Chem.

[27] Chatterjee TK, Fisher RA (2003). Mild heat and proteotoxic stress promote unique subcellular trafficking and nucleolar accumulation of RGS6 and other RGS proteins. Role of the RGS domain in stress-induced trafficking of RGS proteins. J Biol Chem.

[28] Harding SM, Boiarsky JA, Greenberg RA (2015). ATM dependent silencing links nucleolar chromatin reorganization to DNA damage recognition. Cell Rep.

[29] Basak M, Sengar AS, Das K, Mahata T, Kumar M, Kumar D, Biswas S, Sarkar S, Kumar P, Das P, Stewart A, Maity B (2023). A RGS7-CaMKII complex drives myocyte-intrinsic and myocyte-extrinsic mechanisms of chemotherapy-induced cardiotoxicity. Proc Natl Acad Sci U S A.

[30] Das K, Basak M, Mahata T, Kumar M, Kumar D, Biswas S, Chatterjee S, Moniruzzaman M, Saha NC, Mondal K, Kumar P, Das P, Stewart A, Maity B (2022). RGS11-CaMKII complex mediated redox control attenuates chemotherapy-induced cardiac fibrosis. Redox Biol.

[31] Long CS, Henrich CJ, Simpson PC (1991). A growth factor for cardiac myocytes is produced by cardiac nonmyocytes. Cell Regul.

[32] Pramanick A, Chakraborti S, Mahata T, Basak M, Das K, Verma SK, Sengar AS, Singh PK, Kumar P, Bhattacharya B, Biswas S, Pal PB, Sarkar S, Agrawal V, Saha S, Nath D, Chatterjee S, Stewart A, Maity B (2021). G protein beta5-ATM complexes drive acetaminophen-induced hepatotoxicity. Redox Biol.

[33] Chakraborti S, Pramanick A, Saha S, Roy SS, Chaudhuri AR, Das M, Ghosh S, Stewart A, Maity B (2018). Atypical G Protein beta5 promotes cardiac oxidative stress, apoptosis, and fibrotic remodeling in response to multiple cancer chemotherapeutics. Cancer Res.

[34] Kalyanaraman B, Darley-Usmar V, Davies KJ, Dennery PA, Forman HJ, Grisham MB, Mann GE, Moore K, Roberts LJ, Ischiropoulos H (2012). Measuring reactive oxygen and nitrogen species with fluorescent probes: challenges and limitations. Free Radic Biol Med.

[35] Pierce BG, Wiehe K, Hwang H, Kim BH, Vreven T, Weng Z (2014). ZDOCK server: interactive docking prediction of protein-protein complexes and symmetric multimers. Bioinformatics.

[36] Pierce BG, Hourai Y, Weng Z (2011). Accelerating protein docking in ZDOCK using an advanced 3D convolution library. PLoS ONE.

[37] Krieger E, Vriend G (2015). New ways to boost molecular dynamics simulations. J Comput Chem.

[38] Raj R, Agarwal N, Raghavan S, Chakraborti T, Poluri KM, Kumar D (2020). Exquisite binding interaction of 18beta-Glycyrrhetinic acid with histone like DNA binding protein of Helicobacter pylori: A computational and experimental study. Int J Biol Macromol.

[39] Raj R, Agarwal N, Raghavan S, Chakraborti T, Poluri KM, Pande G, Kumar D (2021). Epigallocatechin gallate with potent anti-helicobacter pylori activity binds efficiently to its histone-like DNA binding protein. ACS Omega.

[40] Dickson CJ, Madej BD, Skjevik AA, Betz RM, Teigen K, Gould IR, Walker RC (2014). Lipid14: the amber lipid force field. J Chem Theory Comput.

[41] Hornak V, Abel R, Okur A, Strockbine B, Roitberg A, Simmerling C (2006). Comparison of multiple Amber force fields and development of improved protein backbone parameters. Proteins.

[42] Negi SS, Schein CH, Oezguen N, Power TD, Braun W (2007). InterProSurf: a web server for predicting interacting sites on protein surfaces. Bioinformatics.

[43] Bicknell K, Brooks G, Kaiser P, Chen H, Dove BK, Hiscox JA (2005). Nucleolin is regulated both at the level of transcription and translation. Biochem Biophys Res Commun.

[44] Bonnet H, Filhol O, Truchet I, Brethenou P, Cochet C, Amalric F, Bouche G (1996). Fibroblast growth factor-2 binds to the regulatory beta subunit of CK2 and directly stimulates CK2 activity toward nucleolin. J Biol Chem.

[45] Henras AK, Plisson-Chastang C, O’Donohue MF, Chakraborty A, Gleizes PE (2015). An overview of pre-ribosomal RNA processing in eukaryotes. Wiley Interdiscip Rev RNA.

[46] Jia L, Linder ME, Blumer KJ (2011). Gi/o signaling and the palmitoyltransferase DHHC2 regulate palmitate cycling and shuttling of RGS7 family-binding protein. J Biol Chem.

[47] Song JH, Waataja JJ, Martemyanov KA (2006). Subcellular targeting of RGS9-2 is controlled by multiple molecular determinants on its membrane anchor, R7BP. J Biol Chem.

[48] Drenan RM, Doupnik CA, Jayaraman M, Buchwalter AL, Kaltenbronn KM, Huettner JE, Linder ME, Blumer KJ (2006). R7BP augments the function of RGS7*Gbeta5 complexes by a plasma membrane-targeting mechanism. J Biol Chem.

[49] Drenan RM, Doupnik CA, Boyle MP, Muglia LJ, Huettner JE, Linder ME, Blumer KJ (2005). Palmitoylation regulates plasma membrane-nuclear shuttling of R7BP, a novel membrane anchor for the RGS7 family. J Cell Biol.

[50] Martemyanov KA, Yoo PJ, Skiba NP, Arshavsky VY (2005). R7BP, a novel neuronal protein interacting with RGS proteins of the R7 family. J Biol Chem.

[51] Liapis E, Sandiford S, Wang Q, Gaidosh G, Motti D, Levay K, Slepak VZ (2012). Subcellular localization of regulator of G protein signaling RGS7 complex in neurons and transfected cells. J Neurochem.

[52] Huang J, Stewart A, Maity B, Hagen J, Fagan RL, Yang J, Quelle DE, Brenner C, Fisher RA (2014). RGS6 suppresses Ras-induced cellular transformation by facilitating Tip60-mediated Dnmt1 degradation and promoting apoptosis. Oncogene.

[53] Rubbi CP, Milner J (2003). Disruption of the nucleolus mediates stabilization of p53 in response to DNA damage and other stresses. EMBO.

[54] Espada J, Ballestar E, Santoro R, Fraga MF, Villar-Garea A, Nemeth A, Lopez-Serra L, Ropero S, Aranda A, Orozco H, Moreno V, Juarranz A, Stockert JC, Langst G, Grummt I, Bickmore W, Esteller M (2007). Epigenetic disruption of ribosomal RNA genes and nucleolar architecture in DNA methyltransferase 1 (Dnmt1) deficient cells. Nucleic Acids Res.

[55] Koiwai K, Noma S, Takahashi Y, Hayano T, Maezawa S, Kouda K, Matsumoto T, Suzuki M, Furuichi M, Koiwai O (2011). TdIF2 is a nucleolar protein that promotes rRNA gene promoter activity. Genes Cells.

[56] Halkidou K, Logan IR, Cook S, Neal DE, Robson CN (2004). Putative involvement of the histone acetyltransferase Tip60 in ribosomal gene transcription. Nucleic Acids Res.

[57] Basak M, Das K, Mahata T, Kumar D, Nagar N, Poluri KM, Kumar P, Das P, Stewart A, Maity B (2023). RGS7 balances acetylation/de-acetylation of p65 to control chemotherapy-dependent cardiac inflammation. Cell Mol Life Sci.

[58] Das S, Cong R, Shandilya J, Senapati P, Moindrot B, Monier K, Delage H, Mongelard F, Kumar S, Kundu TK, Bouvet P (2013). Characterization of nucleolin K88 acetylation defines a new pool of nucleolin colocalizing with pre-mRNA splicing factors. FEBS Lett.

[59] Ko CY, Lin CH, Chuang JY, Chang WC, Hsu TI (2018). MDM2 degrades deacetylated nucleolin through ubiquitination to promote glioma stem-like cell enrichment for chemotherapeutic resistance. Mol Neurobiol.

[60] Stewart A, Maity B, Anderegg SP, Allamargot C, Yang J, Fisher RA (2015). Regulator of G protein signaling 6 is a critical mediator of both reward-related behavioral and pathological responses to alcohol. Proc Natl Acad Sci U S A.

[61] Das K, Basak M, Mahata T, Biswas S, Mukherjee S, Kumar P, Moniruzzaman M, Stewart A, Maity B (2023). Cardiac RGS7 and RGS11 drive TGFbeta1-dependent liver damage following chemotherapy exposure. FASEB J.

[62] Maity B, Stewart A, O'Malley Y, Askeland RW, Sugg SL, Fisher RA (2013). Regulator of G protein signaling 6 is a novel suppressor of breast tumor initiation and progression. Carcinogenesis.

[63] Yang J, Platt LT, Maity B, Ahlers KE, Luo Z, Lin Z, Chakravarti B, Ibeawuchi SR, Askeland RW, Bondaruk J, Czerniak BA, Fisher RA (2016). RGS6 is an essential tumor suppressor that prevents bladder carcinogenesis by promoting p53 activation and DNMT1 downregulation. Oncotarget.

[64] Yang J, Huang J, Maity B, Gao Z, Lorca RA, Gudmundsson H, Li J, Stewart A, Swaminathan PD, Ibeawuchi SR, Shepherd A, Chen CK, Kutschke W, Mohler PJ, Mohapatra DP, Anderson ME, Fisher RA (2010). RGS6, a modulator of parasympathetic activation in heart. Circ Res.

[65] Posokhova E, Wydeven N, Allen KL, Wickman K, Martemyanov KA (2010). RGS6/Gbeta5 complex accelerates IKACh gating kinetics in atrial myocytes and modulates parasympathetic regulation of heart rate. Circ Res.

[66] Nolte IM, Munoz ML, Tragante V, Amare AT, Jansen R, Vaez A, Von Der Heyde B, Avery CL, Bis JC, Dierckx B, Van Dongen J (2017). Genetic loci associated with heart rate variability and their effects on cardiac disease risk. Nat Commun.

[67] Posokhova E, Ng D, Opel A, Masuho I, Tinker A, Biesecker LG, Wickman K, Martemyanov KA (2013). Essential role of the m2R-RGS6-IKACh pathway in controlling intrinsic heart rate variability. PLoS ONE.

[68] Monte E, Mouillesseaux K, Chen H, Kimball T, Ren S, Wang Y, Chen JN, Vondriska TM, Franklin S (2013). Systems proteomics of cardiac chromatin identifies nucleolin as a regulator of growth and cellular plasticity in cardiomyocytes. Am J Physiol Heart Circ Physiol.

[69] Mariero LH, Torp MK, Heiestad CM, Baysa A, Li Y, Valen G, Vaage J, Stenslokken KO (2019). Inhibiting nucleolin reduces inflammation induced by mitochondrial DNA in cardiomyocytes exposed to hypoxia and reoxygenation. Br J Pharmacol.

