# Supplementary material for: RGS6 drives cardiomyocyte death following nucleolar stress by suppressing Nucleolin/miRNA-21
Source: J Transl Med. 2024 Feb 26;22:204. doi: 10.1186/s12967-024-04985-3 (PMC10895901; doi:10.1186/s12967-024-04985-3)
Supplement: Supplementary file 1 — Additional file 1: Figure S1. Supplemental molecular modeling of the RGS6-Nucleolin complex. Figure S2. RSG6 modules expression of Nucleolin and Nucleophosmin in human cardiomyocytes. Figure S3. RGS6 promotes nucleolar stress-driven cell death by down-regulating Nucleolin in human myocytes. Figure S4. RGS6 controls expression of miRNA-21 and target genes in murine VCM. Figure S5. Chemotherapy alters expression of miRNA-21 target genes in human and murine myocardium. Figure S6. RGS6 drives changes in nucleolar function in murine VCM. Figure S7. Inhibition of miRNA-21 or Nucleolin depletion phenocopies the impact of RGS6 on nucleolar stress in human cardiomyocytes. Table S1. Reagent List. TableS2. Cell Line List. Table S3. Antibody List. Table S4. Assay Kit List. Table S5. Primer List. Table S6. The residues undergoing significant change in the solvent accessible surface area (SASA) upon complex formation between RGS6 and Nucleolin. Table S7. Clinical Data for chemotherapy-treated heart autopsy samples: controls (Con) 1-12; chemotherapy patients without detectable fibrosis (C-F) 1-8; and chemotherapy patients with detectable fibrosis (C+F) 1-8. [file 12967_2024_4985_MOESM1_ESM.docx]

**Data Additional**

**Additional Methods**

*Exact numbers of animals used*:

Main figure 1: 1D, 8 mice (4 males and 4 females, age matched 24-28 weeks old) were used to isolate VCM and divided the cells into three groups and each group has 8 replicates. 1E,samplesfrom the same population of cells from experiment 1D were separated for RNA isolation and qPCR studies. 1F, cells from experiment 1D were also used for protein expression studies.Cells were divided for each treatment group and run in replicates of 3-6 as noted in the figure legends.

Main figure 2: 2A-C, Cells isolated for experiment 1D were also used for experiment 2A-C experiment divided again for protein and RNA analyses. 2D-I, 4 adult (2 males and 2 females, age matched 24-28 weeks old) mice were sacrificed to isolate VCM and the respective biochemical assays were performed in 48 well set up with five replicates for each of the four groups. For 2H and 2I experiment, the cells were subjected to RNA analysis (three replicates) and protein expression analysis (six replicates) respectively.

Main figure 4: 24 adult mice (12 males and 12 females, age matched 24-28 weeks old) were divided into two groups as mentioned in experiments 4A & 4E (half with RGSOE and half with respective vector control). For experiments 4B-4D, three mice samples were chosen in a blinded fashion from the above-mentioned group for RNA analysis.

Main figure 5: 5C-5D, 24 mice (age matched 24-28 weeks old) were divided into two groups (ten control with vehicle treated and fourteen with doxorubicin treatment).

Main figure 7: 8 (4 males and 4 females, age matched 24-28 weeks old) mice were used to isolate VCM and cells were divided into four groups for RNA analysis, apoptosis, and cell viability experiments (7A-7D). Isolated cells were also used for experiments 7E-7H (24 well plate set up for 7E & 7F and 48 well plate set up for 7G & 7H).

Main figure 8: 24 mice (12 males and 12 females, age matched 24-28 weeks old) were divided into four groups and protein analysis were performed using heart lysates (8A & 8F). For experiments 8B-8E, three mouse samples from each group were chosen in a blinded fashion for RNA analysis.

Supplementary figure S4: S4A-S4F mice tissue from 2I experiment was used for RNA analysis.

Supplementary figure S5: S5B mice heart lysates were used for protein analysis from the experiment in figure 5.

Supplementary figure S6: 6 mice (3 males and 3 females, age matched 24-28 weeks old) were used to isolate VCM and four groups were used for experiment S6A-S6D.

A total of 98 mice (49 males and 49 females, age matched) were used for the study.

**Additional Figures**

**
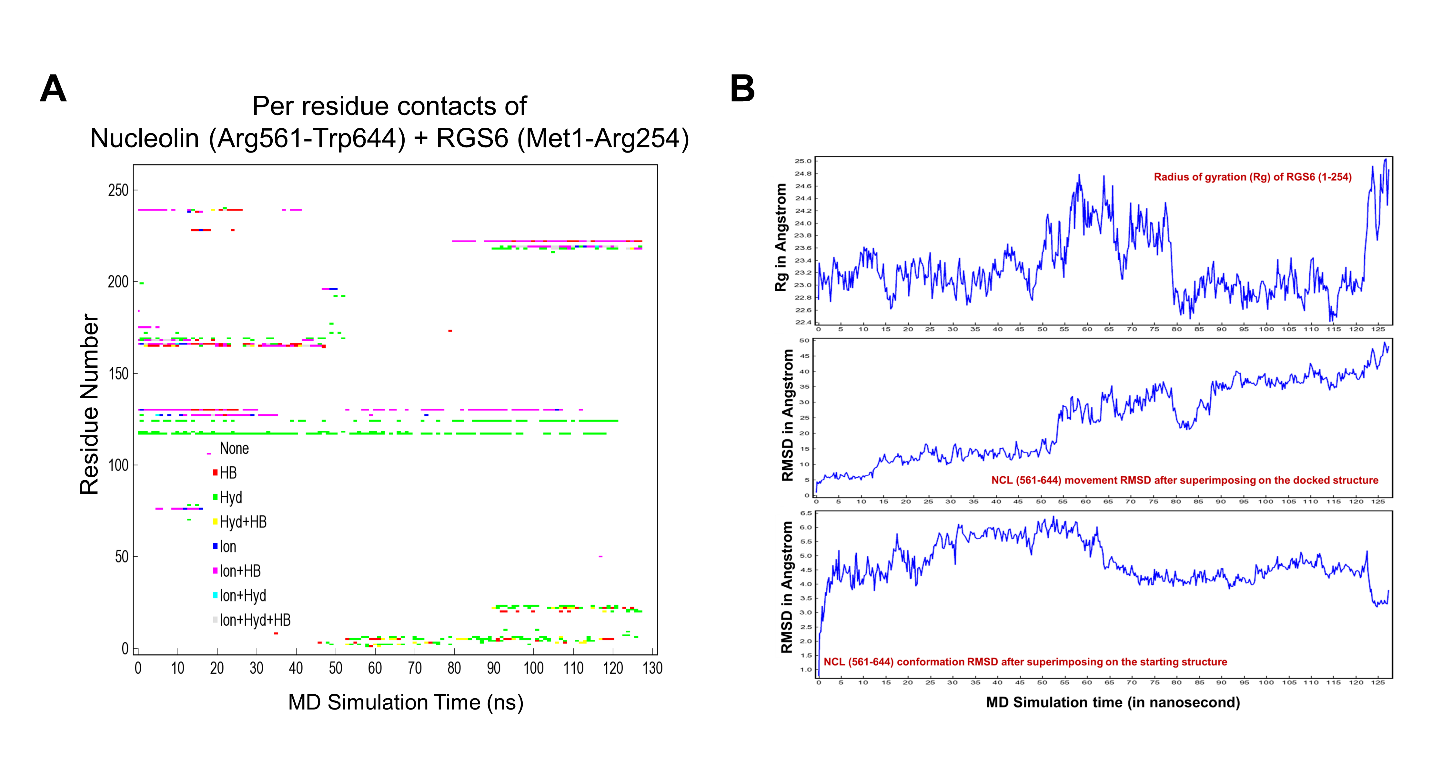
**

**Figure S1** – Supplemental molecular modeling of the RGS6-Nucleolin complex. The molecular protein-protein interaction between RGS6 (1-472) and human Nucleolinprotein (Gly300-Trp644) was established via*in-silico* molecular docking using the ZDOCK webserver application (<https://zdock.umassmed.edu>). (A) The solution stability of the modelled RGS6-Nucleolin complex evaluated based on per-residue contacts of RGS6 (Met1-Arg254) with Nucleolin (Met1-Arg254)as a function of simulation time (in nanoseconds, ns). (B) Radius of gyration (Rg) of RGS6 (1-254) [top], Nucleolin (NCL) movement root mean square distance (RMSD) after superimposing on the docked structure [middle], andNucleolin conformation RMSD after superimposing on the starting structure as a function of simulation time [bottom].


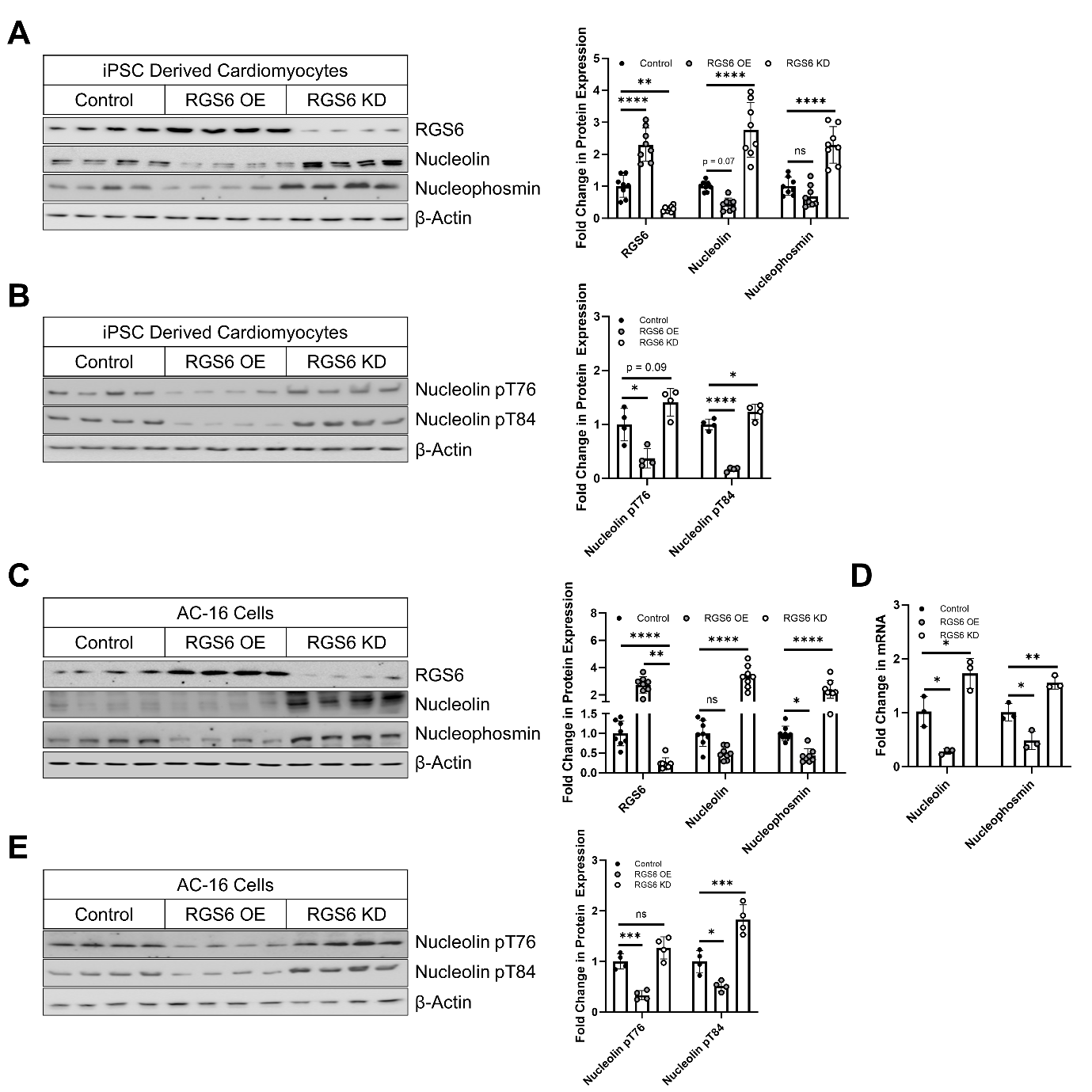


**Figure S2** – RSG6 modules expression of Nucleolin and Nucleophosmin in human cardiomyocytes. (A-B) human induced pluripotent stem cell (iPSC)-derived cardiomyocytes were transduced with RGS6-pEGFP (RGS6 OE), RGS6 specific shRNA (RGS6 KD) or vector control. (A) Immunoblots and quantification for RGS6, Nucleolin, and Nucleophosmin (n=8). (B) Immunoblots and quantification for Nucleolin pT76andNucleolin pT84 (n=4). (C-E) Human AC-16 cardiomyocytes were transduced with RGS6-pEGFP (RGS6 OE), RGS6 specific shRNA (RGS6 KD) or vector control. (C) Immunoblots with quantification for RGS6, Nucleolin, and Nucleophosmin (n=8). (D) Fold change inNucleolin and Nucleophosmin mRNA (n=3). (E) Immunoblots with quantification for Nucleolin pT76andNucleolin pT84.Β-Actin served as the loading control for immunoblots (n=4).Data were analyzed by one-way ANOVA with Sidak’s post-hoc test. **P <*0.05, ***P <*0.01, ****P <*0.001, *****P <*0.0001. ns = not significant. Data are presented as mean ± SEM.


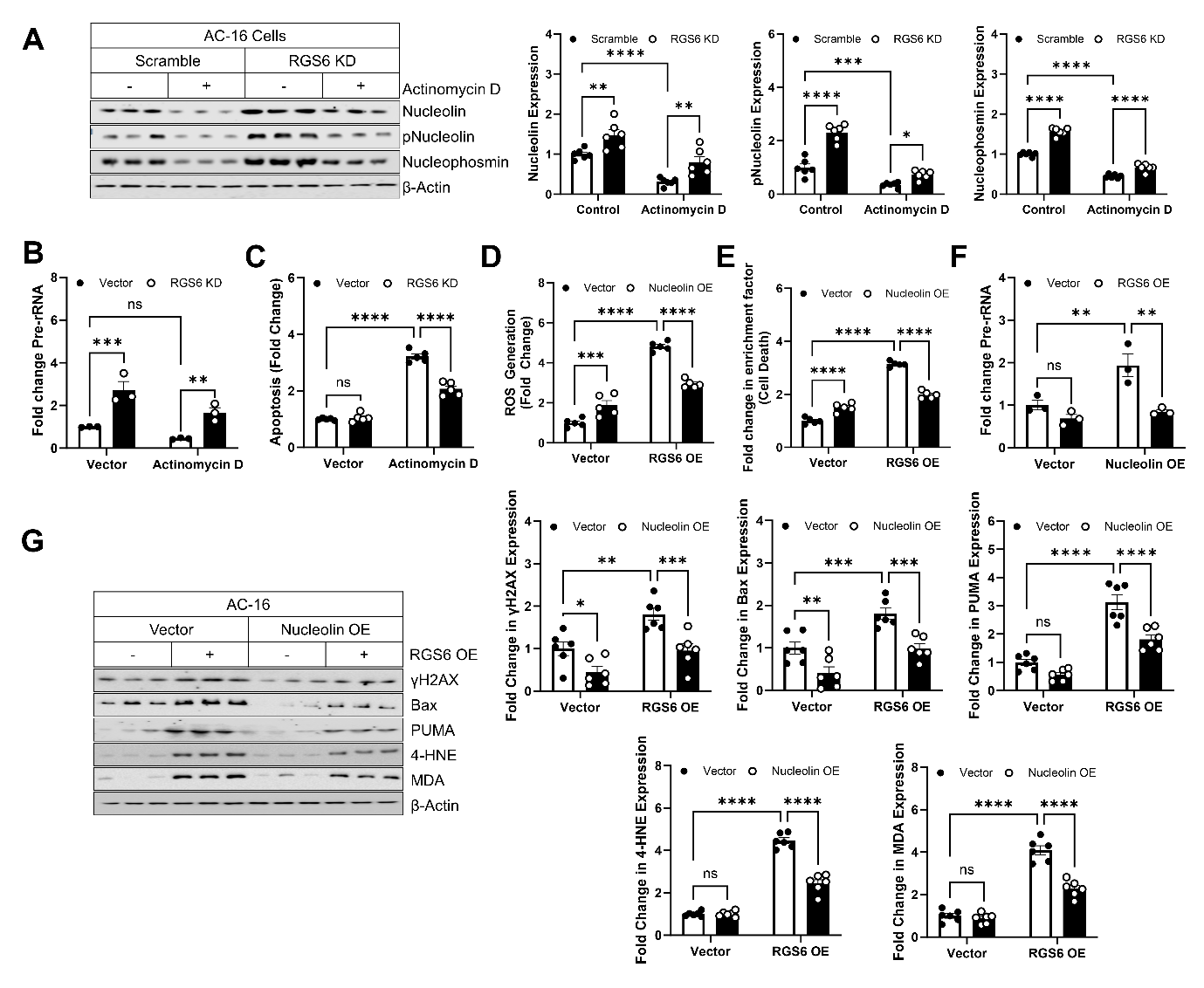


**Figure S3** –RGS6 promotes nucleolar stress-driven cell death by down-regulating Nucleolin in human myocytes. (A-C) Human AC-16 cardiomyocytes were transduced with scramble or RGS6 specific shRNA (RGS6 KD) ±Actinomycin D (5µM, 16 hours). (A) Representative immunoblots and quantifications for Nucleolin, pNucleolin (Nucleolin pT76), and Nucleophosmin (n=6). (B) Fold change inpre-rRNA (n=3). (C) Apoptosis (cytoplasmic histone-associated DNA fragments; n=5). (D-E) Human AC-16 cardiomyocyteswere transduced with Nucleolin-HA (Nucleolin OE), RGS6-pEGFP (RGS6 OE), both constructs, or vector control and harvested36 hours after transduction. (D) CM-H_2_-DCFDA fluorescence (ROS; n=5). (E) Apoptosis (cytoplasmic histone-associated DNA fragments; n=5). (F) Fold change inpre-rRNA (n=3). (G) Representative immunoblots with quantification for γH2AX, Bax, PUMA, 4-HNE, and MDA.β-Actin served as the loading control for immunoblots (n=6).Data were analyzed by two-way ANOVA with Sidak’s post-hoc test. **P <*0.05, ***P <*0.01, ****P <*0.001, *****P <*0.0001. ns = not significant. Data are presented as mean ± SEM.


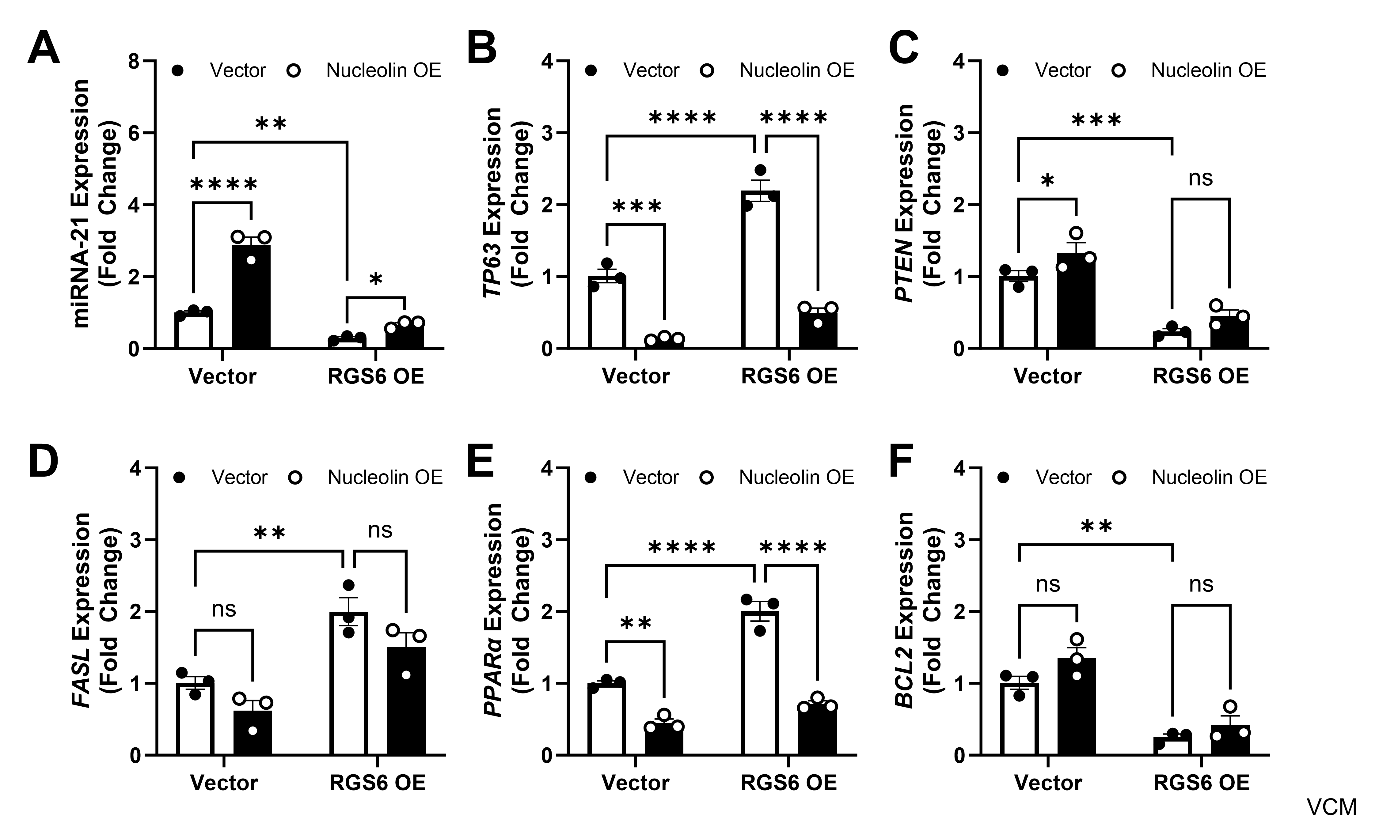


**Figure S4** –RGS6 controls expression of miRNA-21 and target genes in murine VCM. Murine VCMs were transduced with Nucleolin-HA (Nucleolin OE), RGS6-pEGFP (RGS6 OE), both constructs, or vector control and harvested36 hours after transduction. (A) Fold change in miRNA-21 level (n=3). (B-F) mRNA expression of miRNA-21 target genes (B) *TP63* (C) *PTEN* (D) *FASL* (E) *PPARα*, and (F) *BCL2* (n=3). Data were analyzed by two-way ANOVA with Sidak’s post-hoc test. **P <*0.05, ***P <*0.01, ****P <*0.001, *****P <*0.0001. ns = not significant. Data are presented as mean ± SEM.


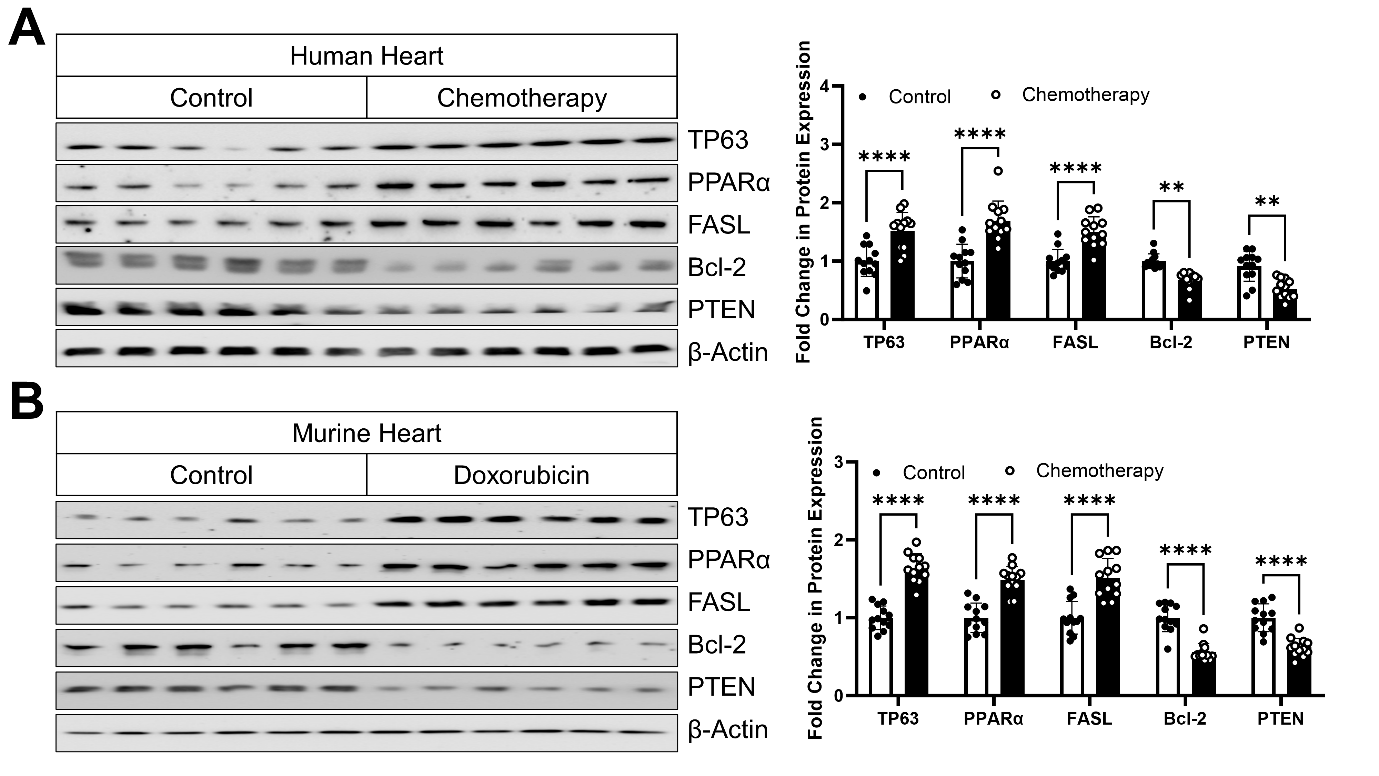


**Figure S5** –Chemotherapy alters expression of miRNA-21 target genes in human and murine myocardium. (A)Representative immunoblots and quantification of cardiac expression of miRNA-21 targets TP63, PPARα, FASL, Bcl-2, and PTEN in controls and patients with a history of chemotherapy (n=12).(B) Representative immunoblots and quantification of cardiac expression of miRNA-21 targets TP63, PPARα, FASL, Bcl-2, and PTEN in murine heart treated with doxorubicin (cumulative dose of 45 mg/kg 9 mg/kg, i.p. every other week) or saline for 10 weeks. Samples were collected 1 week after mice received last dose later for biochemical and histological analyses (n=12).Β-Actin served as the loading control for immunoblots.Data were analyzed by two-tailed student’s t-test. **P <*0.05, ***P <*0.01, ****P <*0.001, *****P <*0.0001. ns = not significant. Data are presented as mean ± SEM.


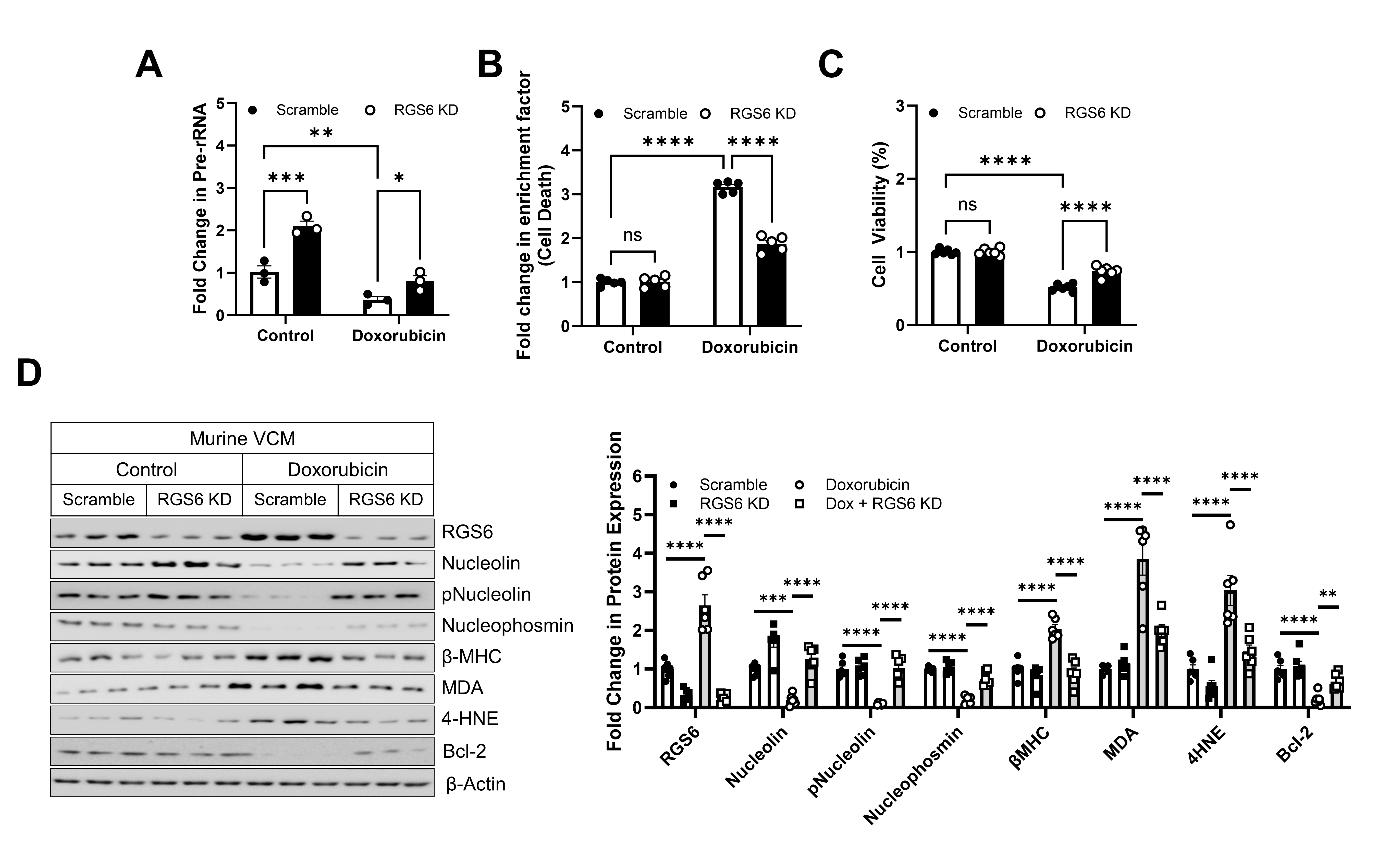


**Figure S6** –RGS6 drives changes in nucleolar function in murine VCM. Murine VCM were transduced with scramble or RGS6 specific shRNA (RGS6 KD) ± doxorubicin (3 μM, 18 hours). (A) Fold change inpre-rRNA (n=3). (B) Apoptosis (cytoplasmic histone-associated DNA fragments; n=5). (C) MTT assay to determine cell viability (n=6). (D) Representative immunoblots and quantification for RGS6, Nucleolin, pNucleolin (pT76), Nucleophosmin, β-MHC, MDA, 4-HNE, and Bcl-2 (n=6).Β-Actin served as the loading control for immunoblots. Data were analyzed by two-way ANOVA with Sidak’s post-hoc test. **P <*0.05, ***P <*0.01, ****P <*0.001, *****P <*0.0001. ns = not significant. Data are presented as mean ± SEM.


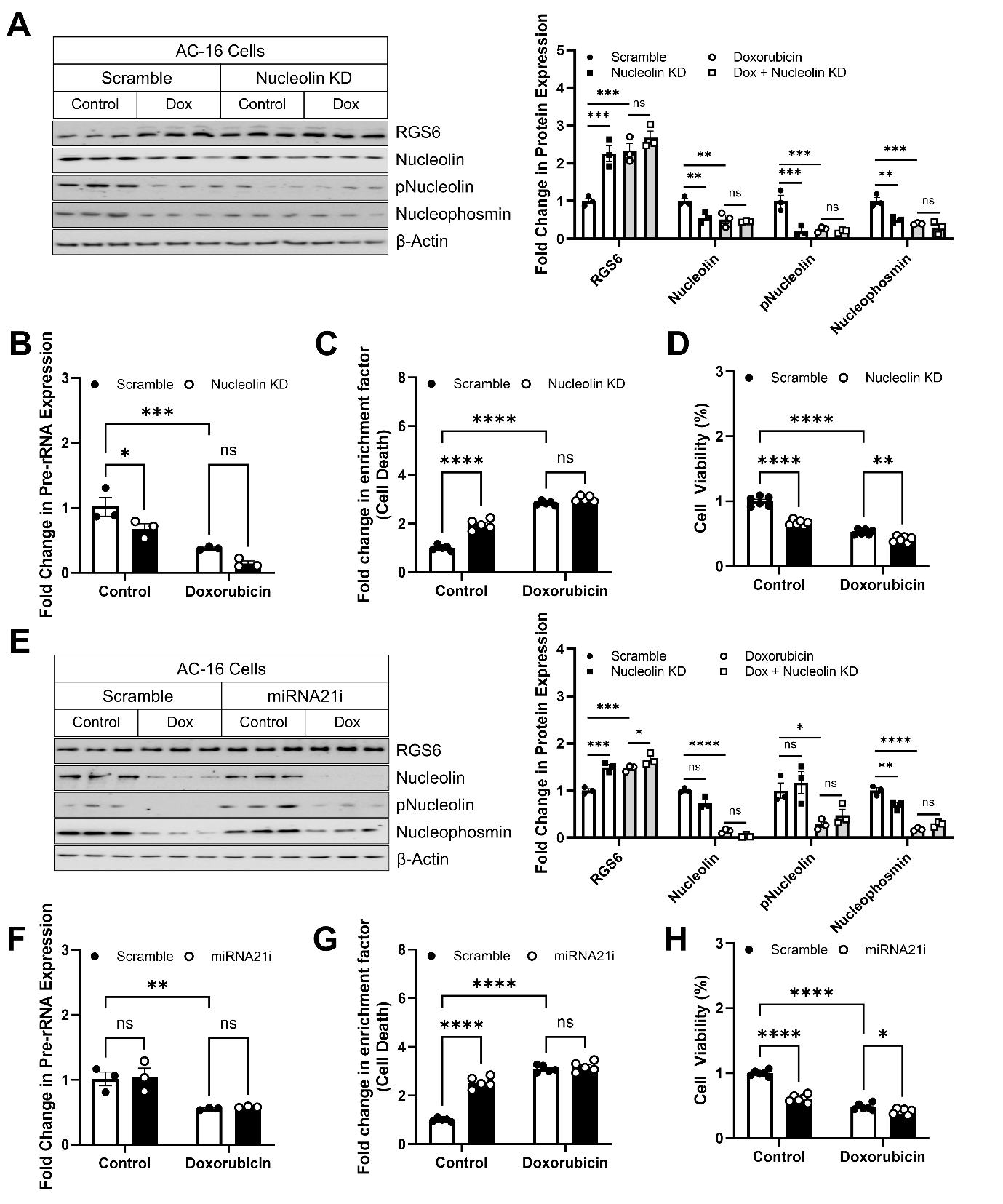


**Figure S7** –Inhibition of miRNA-21 or Nucleolin depletion phenocopies the impact of RGS6 on nucleolar stress in human cardiomyocytes. (A-D) Human AC-16 cardiomyocytes were transduced with scramble or Nucleolin specific shRNA (Nucleolin KD) ± doxorubicin (3 μM, 18 hours). (A) Immunoblots for RGS6, Nucleolin,pNucleolin (pT76),andNucleophosmin (n=3). (B) Fold change inPre-rRNA(n=3). (C) Apoptosis (cytoplasmic histone-associated DNA fragments; n=5). (D) MTT assay to determine cell viability (n=6). (E-H) Human AC-16 cardiomyocytes were transduced with scramble or miRNA-21 inhibitor (miRNA-21i) ± doxorubicin (3 μM, 18 hours). (E) Immunoblots and quantification for RGS6, Nucleolin, pNucleolin (pT76), Nucleophosmin (n=3). (F) Fold change inPre-rRNA (n=3). (G) Apoptosis (cytoplasmic histone-associated DNA fragments; n=5). (H) MTT assay to determine cell viability (n=6). β-Actin served as the loading control for immunoblots. Data were analyzed by two-way ANOVA with Sidak’s post-hoc test. **P <*0.05, ***P <*0.01, ****P <*0.001, *****P <*0.0001. ns = not significant. Data are presented as mean ± SEM.

**Additional Tables**

**Table S1**: Reagent List

| **Company** | **Location** | **Reagent** |
| --- | --- | --- |
| Sigma | St. Louis, MO, USA | Actinomycin D |
|  |  | CM-H_2_DCFDA |
| Addgene | Watertown, MA, USA | pLenti CMV Puro DEST cloning vector |
|  |  | pMD2.G VSV-G envelope expressing plasmid |
|  |  | psPAX2 |
|  |  | Protein A/G sepharose |
|  |  | Doxorubicin |
|  |  | Thiazolyl blue tetrazolium bromide (MTT) |
|  |  | 10X RIPA |
|  |  | 50X DAB Chromogen& DAB Substrate |
|  |  | ECL Detection kit |
|  |  | Bradford reagent |
|  |  | Protease inhibitor cocktail |
|  |  | Phosphatase inhibitor cocktail |
| Thermo Fisher Scientific | Waltham, MA, USA | Phusion Hot Start II High-Fidelity PCR Master Mix (F-565S) |
|  |  | Platinum^TM^ Super FiII PCR Master Mix |
|  |  | FBS (Gibco) |
|  |  | RNA later |
|  |  | Trypsin EDTA 0.05% and 0.25% |
|  |  | Alexafluor conjugates |
|  |  | DNA Ladder (50, 100, 1000 kb) |
|  |  | TaqManRNAase |
|  |  | OptiMEM, MEM, DMEM |
|  |  | Glycogen |
|  |  | Dream Taq |
| Invitrogen | Carlsbad, CA, USA | Superscript III RT/Platinum Taq Mix |
|  |  | Lipofectamine 3000 |
|  |  | Invivofectamine 3.0 |
|  |  | Platinum Taq DNA Polymerase High Fidelity |
|  |  | Verso cDNA synthesis Kit |
| Takara Bio | Kyoto, Japan | pMD20-T vector |
| Sisco Research Laboratory | Mumbai, India | Acrylamide |
|  |  | Aprotinin |
|  |  | PMSF |
|  |  | RNAase inhibitor |
|  |  | Protein ladder |
|  |  | 6X loading dye |
|  |  | Ethidium bromide |
|  |  | DTT |
|  |  | 50X TAE |
|  |  | Bovine Serum Albumin |
|  |  | DMSO |
|  |  | Ponceau Red Stain |
|  |  | Xylene |
|  |  | 4% Formaldehyde |
| Himedia | Mumbai, India | Puromycinedihydrochloride |
|  |  | Collagenase type II & type IV |
|  |  | Ampicillin sodium |
|  |  | Proteinase K |
|  |  | Amphotericine B |
|  |  | PBS |
|  |  | D-Mannitol |
| New England Biolabs | Ipswich, MA, USA | Restriction Enzymes: XhoI, HindIII-HF,EcoRI-HF,KpnI |
| Santa Cruz Biotechnology | Dallas, TX, USA | shRNA for RGS6, Nucleolin |
| Biorad | USA | Syber green master mix |
| Accegen Biotechnology | NJ, USA | miRNA21 inhibitor (m,h) |

**TableS2**: Cell Line List

| Company | Location | Cell Line | Catalog # | Culture Conditions |
| --- | --- | --- | --- | --- |
| Merck & Co. | Kenilworth, NJ, USA | AC-16 | SCC109 | 37°C incubator at 5% CO_2_ in DMEM + 10% FBS |
| Takara | Kusatsu,Japan | Cellartiscardiomyocytes (iPSC CM) | ChipSC22 | 37°C incubator at 5% CO_2_ in cellartis CM culture base (Y10063) |

**Table S3**: Antibody List

| **Company** | **Location** | **Antibody** | **Catalog #** | **Dilution** |
| --- | --- | --- | --- | --- |
| Abcam | Cambridge, UK | Nucleolin | ab136649 | WB (1:1000), IHC (1:200), IP (1:100) |
|  |  | Nucleolin pT76 | ab168363 | WB (1:1000) |
|  |  | Nucleolin pT84 | ab155977 | WB (1:1000) |
|  |  | Nucleophosmin | ab10530 | WB (1:1000) |
|  |  | Mouse Secondary-HRP | ab97023 | WB (1:2000), IHC (1:500) |
|  |  | Rabbit Secondary-HRP | ab97051 | WB (1:2000), IHC (1:500) |
|  |  | Cardiac Troponin T | ab8295 | WB (1:1000), IHC (1:500) |
|  |  | 4-HNE | ab46545 | WB (1:1000) |
|  |  | MDA | Ab27642 | WB (1:1000) |
|  |  | RGS6 | ab128943 | WB (1:1000) |
|  |  | ANP | ab189921 | WB (1:1000) |
|  |  | Β-Actin | ab8227 | WB (1:1000) |
|  |  | Bax | ab216494 | WB (1:1000) |
|  |  | PUMA | ab9643 | WB (1:1000) |
|  |  | γH2AX | ab26350 | WB (1:1000) |
|  |  | TP63 | ab124762 | WB (1:1000) |
|  |  | Anti-HA | ab9110 | WB (1:1000) |
| Cell Signaling Technology | Danvers, MA, USA | Bcl2 | CST- 2807P | WB (1:1000) |
|  |  | GFP | 2956 | WB (1:1000), IP (1:200) |
|  |  | PTEN | 9552 | WB (1:1000) |
|  |  | β-MHC | MA1-26180 | WB (1:1000) |
| Thermo Fisher Scientific | Waltham, MA, USA | FASL | 356-MSM6-P1 | WB (1:1000) |
| Invitrogen |  | PPARα | PA1-822A | WB (1:800) |

**Table S4**: Assay Kit List

| **Company** | **Location** | **Assay** | **Catalog #** |
| --- | --- | --- | --- |
| Abcam | Cambridge, UK | Ca^2+^ Flux Assay Kit | ab102505 |
|  |  | Mitochondrial isolation Kit | ab110170 |
|  |  | Mitochondrial Membrane Potential Assay Kit | ab113852 |
| Roche | San Francisco, CA, USA | Cell Death Detection Kit | C755B93 |

**Table S5:** Primer List

H = human; M = mouse

| **#** | **Names of Primer** | **Sequence of Primer** |
| --- | --- | --- |
| 1 | Nucleolin H Sense EcoRIpCMV-HA-N | 5’ATAGAATTCATGGTGAAGCTCGCGAAGGC3’ |
| 2 | Nucleolin H Antisense XhoIpCMV-HA-N | 3’ATACTCGAGCTATTCAAACTTCGTCTTCT5’ |
| 3 | Nucleolin M Sense EcoRIpCMV-HA-N | 5’ATAGAATTCATGGTGAAGCTCGCAAAGGCTGGCA3’ |
| 4 | Nucleolin M Antisense XhoIpCMV-HA-N | 3’ATACTCGAGCTATTCAAACTTCGTCTTCTTT5’ |
| 5 | miRNA21RNAi H Sense XhoIpmR-ZsGreen1 | 5’ATACTCGAGTAAGTGTTTTATTCTTAGTG3’ |
| 6 | miRNA21 RNAi H Antisense HindIIIpmR-ZsGreen1 | 3’ATAAAGCTTTTATTTGTGGTCATGAAGAC5’ |
| 7 | miRNA21 RNAi M Sense XhoIpmR-ZsGreen1 | 5’ATACTCGAGGGCTTTTTCATGTTCATGTTAGT3’ |
| 8 | miRNA21 RNAi M Antisense KpnIpmR-ZsGreen1 | 3’ATAGGTACCCCTTTCCAAAATCCATGAGGCAA5’ |
| 9 | RGS6 H SenseXhoI pEGFP-N1 | 5’ATACTCGAGATGGCTCAAGGATCCGG3’ |
| 10 | RGS6 H Antisense HindIII pEGFP-N1 | 5’ATAAAGCTTGGAGGACTGCATCAGGC3’ |
| 11 | RGS6 M Sense XhoI pEGFp-N1 | 5’ATACTCGAGATGGCTCAGGGGTCCGGGGAC3’ |
| 12 | RGS6 M Antisense HindIII pEGFP-N1 | 3’ATAAAGCTTGGAGGACTGCATCAGGCCCGT5’ |
| 13 | NCL RT H Sense | 5'GATCGATGGGCGATCTATTTCC3' |
| 14 | NCL RT H Antisense | 3'TTCACCACTCCAAGTGCTATTC5' |
| 15 | NPM RT H Sense | 5’ACTTAGTAGCTGTGGAGGAAGA3’ |
| 16 | NPM RT H antisense | 3’GCAGACCGCTTTCCAGATATAC5’ |
| 17 | RGS6 RT H Sense | 5’AGGACATCCGGAAACAGATAAC3’ |
| 18 | RGS6 RT H Antisense | 3’TTGTTCCGTGTAGGCAATTAAAC5’ |
| 19 | NCL RT M Sense | 5’GACGCCATGGAGATCAGATTAG3’ |
| 20 | NCL RT M Antisense | 3’CTCTGCATCAGCTTCAGACTT5’ |
| 21 | NPM RT M Sense | 5’AACTCTTAGGCATGTCTGGAAA3’ |
| 22 | NPM RT M Antisense | 3’CCTCATCATCGTCCTCATCATC  5’ |
| 23 | RGS6 RT M Sense | 5’GTGGAGTACGACCCATTCATAAC 3’ |
| 24 | RGS6 RT M Antisense | 3’GGGCTCTTTGCTCATCTCTATG5’ |
| 25 | U6SnRNA RT H Sense | 5'CTCGCTTCGGCAGCACA3' |
| 26 | U6SnRNART H Antisense | 3'AACGCTTCACGAATTTGCGT5' |
| 27 | U6SnRNA RT M Sense | 5’CTGAGCATTTGGGAGGTAGAG3’ |
| 28 | U6SnRNA RT M Antisense | 3’GGAATATCTCGGGCTATCTTTGT5’ |
| 29 | miRNA21 H RT Sense | 5’TGTCGGGTAGCTTATCAG3’ |
| 30 | miRNA21 H RT Antisense | 3'TGTCAGACAGCCCATCGAC5' |
| 31 | TP63 H RT Sense | 5’CAGAGTGTGCTGGTACCTTATG3’ |
| 32 | TP63 H RT Antisense | 3’GGTTCATCCCTCCAACACAA5’ |
| 33 | Bcl2 H RT Sense | 5’GGAGGATTGTGGCCTTCTTT3’ |
| 34 | Bcl2 H RT Antisense | 3’GTTCAGGTACTCAGTCATCCAC5’ |
| 35 | PTEN H RT Sense | 5’CGTTACCTGTGTGTGGTGATA3’ |
| 36 | PTEN H RT Antisense | 3 CTCTGGTCCTGGTATGAAGAATG’5’ |
| 37 | PPAR alpha H RT Sense | 5’TGAACTGAGGGACAGTGATTTC 3’ |
| 38 | PPAR alpha H RT Antisense | 3’ CCCAAGGGTAGCTCAGTTTATC5’ |
| 39 | FASLG H RT Sense | 5’CATTTAACAGGCAAGTCCAACTC |
| 40 | FASLG H RT Antisense | 3’CACAAGGCCACCCTTCTTAT 5’ |
| 41 | TP63 M RT Sense | 5’GCATCAGAAAGCAGCAAGTATC3’ |
| 42 | TP63 M RT Antisense | 3’GTCATCTGGATTCCGTGTGTAT5’ |
| 43 | Bcl2 M RT Sense | 5’GGAGGATTGTGGCCTTCTTT3’ |
| 44 | Bcl2 M RT Antisense | 3’GTTCAGGTACTCAGTCATCCAC5’ |
| 45 | PTEN M RT Sense | 5’CTGCCAGCTAAAGGTGAAGATA3’ |
| 46 | PTEN M RT Antisense | 3’ATGGCTGAGGGAACTCAAAG5’ |
| 47 | PPAR alpha M RT Sense | 5’CTGTCGGGATGTCACACAAT3’ |
| 48 | PPAR alpha M RT Antisense | 3’CAGGTCGTGTTCACAGGTAAG5’ |
| 49 | FASLG M RT Sense | 5’TGGGTTGTACTTCGTGTATTCC3’ |
| 50 | FASLG M RT Antisense | 3’CAACCTCTTCTCCTCCATTAGC5’ |
| 51 | miRNA21 M RT Sense | 5’TACCACCTTGTCGGATAGCTTA3’ |
| 52 | miRNA21 M RT Antisense | 3’ AAATGTCAGACAGCCCATCG5’ |
| 53 | ITS1 H RT Sense | 5’CCTCCCCCGCCTCCTCGTCC3’ |
| 54 | ITS1 H RT Antisense | 3’GCCCTCCCGACGGGACTCCC5’ |
| 55 | ITS1 M RT Sense | 5’TCCGTGTCTACGAGGGGCGG3’ |
| 56 | ITS1 MRT Antisense | 3’GGGTGCCGGGAGAGCAAAGC5’ |
| 57 | 18S H Sense | 5’GCCGCTAGAGGTGAAATTCT3’ |
| 58 | 18S H Antisense | 3’TCGGAACTACGACGGTATCT5’ |
| 59 | 18S M Sense | 5’CGAGCCGCCTGGATACC3’ |
| 60 | 18S M Antisense | 3’CATGGCCTCAGTTCCGAAAA5’ |

**Table S6**– The residues undergoing significant change in the solvent accessible surface area (SASA) upon complex formation between RGS6 and Nucleolin. Residues undergoing the greatest shift upon binding are in bold.

| **RGS6 Protein Chain A (1-472)** | | | | |
| --- | --- | --- | --- | --- |
| **Amino Acid Residue** | **Residue Number** | **Monomer Area** | **Complex Area** | **Difference** |
| ILE | 133 | 17.36 | 7.27 | 10.09 |
| GLU | 239 | 172.87 | 158.95 | 13.92 |
| LYS | 179 | 110.51 | 96.42 | 14.09 |
| ASP | 130 | 60.3 | 45.67 | 14.63 |
| GLU | 196 | 37.53 | 22.55 | 14.98 |
| PHE | 171 | 99.73 | 81.45 | 18.28 |
| LEU | 192 | 64.18 | 38.5 | 25.68 |
| PHE | 118 | 33.58 | 5.96 | 27.62 |
| MET | 1 | 228.01 | 199.88 | 28.13 |
| THR | 129 | 47.55 | 19.32 | 28.23 |
| GLU | 175 | 66.21 | 28.13 | 38.08 |
| GLN | 177 | 63.95 | 22.5 | 41.45 |
| ALA | 176 | 49.77 | 6.17 | 43.6 |
| ARG | 165 | 229.31 | 181.8 | 47.51 |
| **ILE** | **180** | **93** | **38.35** | **54.65** |
| **LYS** | **166** | **125.88** | **62.19** | **63.69** |
| **GLU** | **168** | **122.59** | **40.55** | **82.04** |
| **TYR** | **117** | **138.39** | **53.42** | **84.97** |
| **GLN** | **173** | **91.2** | **1.24** | **89.96** |
| **MET** | **172** | **93.69** | **0.11** | **93.58** |
| **PHE** | **169** | **132.94** | **7.01** | **125.93** |

| **NCL Protein Chain B (300-644)** | | | | |
| --- | --- | --- | --- | --- |
| **Amino Acid Residue** | **Residue Number** | **Monomer Area** | **Complex Area** | **Difference** |
| ALA | 566 | 103.98 | 92.93 | 11.05 |
| ASP | 603 | 47.08 | 34 | 13.08 |
| PHE | 575 | 23.26 | 6.39 | 16.87 |
| SER | 571 | 37.3 | 18.45 | 18.85 |
| ASN | 565 | 89.71 | 61.43 | 28.28 |
| ASP | 616 | 39.76 | 10.86 | 28.9 |
| VAL | 601 | 37.55 | 5.65 | 31.9 |
| LYS | 572 | 100.42 | 66.74 | 33.68 |
| THR | 606 | 108.26 | 69.34 | 38.92 |
| PHE | 612 | 55.44 | 9.02 | 46.42 |
| **THR** | **573** | **56.52** | **2.65** | **53.87** |
| **LYS** | **610** | **94.03** | **32.48** | **61.55** |
| **ARG** | **597** | **140.68** | **78.28** | **62.4** |
| **ARG** | **567** | **96.8** | **17.76** | **79.04** |
| **PHE** | **614** | **81.01** | **1.95** | **79.06** |
| **ARG** | **599** | **132.73** | **44.59** | **88.14** |
| **GLU** | **605** | **149.34** | **49.8** | **99.54** |
| **ARG** | **604** | **214.77** | **43.95** | **170.82** |

**Table S7** – :Clinical Data for chemotherapy-treated heart autopsy samples: controls (Con) 1-12; chemotherapy patients without detectable fibrosis (C-F) 1-8; and chemotherapy patients with detectable fibrosis (C+F) 1-8. All “chemotherapy patients” had a history of chemotherapy with regimens containing 5-FU, an anthracycline and/or oxaliplatin.

| Patient ID | Age | Sex | | Cancer | Treatment received | | | Co-morbid conditions | Cause of death | | Fibrosis Score | |
| --- | --- | --- | --- | --- | --- | --- | --- | --- | --- | --- | --- | --- |
| Con 1 | 55 | M | | N/A | N/A | | | Arthritis | Kidney failure | | 0 | |
| Con 2 | 54 | M | | N/A | N/A | | | Mild Asthma | Ischemic stroke | | 0 | |
| Con 3 | 62 | F | | N/A | N/A | | | - | Drowning | | 0 | |
| Con 4 | 65 | F | | N/A | N/A | | | - | Road accident | | 0 | |
| Con 5 | 56 | M | | N/A | N/A | | | Diabetes | Haemorrhagic stroke | | 0 | |
| Con 6 | 52 | F | | N/A | N/A | | | Eczema | Hepatolenticular degeneration | | 0 | |
| Con 7 | 71 | M | | N/A | N/A | | | Asthma | Suicide (by hanging) | | 0 | |
| Con 8 | 75 | F | | N/A | N/A | | | Fatty liver | Ischemic stroke | | 0 | |
| Con 9 | 51 | F | | N/A | N/A | | | Anxiety | Suicide (by hanging) | | 0 | |
| Con 10 | 62 | M | | N/A | N/A | | | IBS | Ischemic stroke | | 0 | |
| Con 11 | 55 | F | | N/A | N/A | | | Fatty liver | Ischemic stroke | | 0 | |
| Con 12 | 72 | F | | N/A | N/A | | | - | Road accident | | 0 | |
| C-F1 | 66 | M | | GI | Folfox + Bavacizumab | | | Asthma | Metastasis | | 0 | |
| C-F2 | 54 | F | | Breast | Anthracycline | | | Mild depression | Metastasis | | 0 | |
| C-F3 | 69 | F | | Breast | Anthracycline | | | Eczema | Metastasis | | 0 | |
| C-F4 | 63 | F | GI | | | Fluoropyrimidine | Mild Lupus | | | Metastasis | | 0 |
| C-F5 | 67 | F | Breast | | | Anthracycline | Psoriasis | | | Metastasis | | 0 |
| C-F6 | 75 | F | Breast | | | Anthracycline+ Capecitabine | Mild diabetes | | | Metastasis | | 0 |
| C-F7 | 61 | M | Colorectal | | | Folfox | Mild hypertension | | | Metastasis | | 0 |
| C-F8 | 78 | M | GI | | | Folfox + Bavacizumab | Fatty liver | | | Metastasis | | 0 |
| C+F1 | 61 | F | Colorectal | | | Folfox | Mild diabetes | | | Myocardial infarction | | 2 |
| C+F2 | 57 | F | Breast | | | Anthracycline | Fatty liver | | | Atrial fibrillation | | 2 |
| C+F3 | 63 | F | Breast | | | Anthracycline | Mild depression | | | Myocardial infarction | | 3 |
| C+F4 | 61 | M | GI | | | Fluoropyrimidine | Asthma | | | Myocardial infarction | | 3 |
| C+F5 | 72 | F | Breast | | | Anthracycline | Fatty liver | | | Heart failure | | 3 |
| C+F6 | 71 | F | Breast | | | Anthracycline | Diverticulitis | | | Myocarditis & Heart failure | | 4 |
| C+F7 | 66 | M | Colorectal | | | Folfox | Diabetes | | | Angina & myocardial infarction | | 3 |
| C+F8 | 59 | M | GI | | | Folfox + Bavacizumab | Arthritis | | | Ventricular tachycardia & heart failure | | 3 |
